# Life Cycle Impacts and Benefits of Wood along the Value Chain: The Case of Switzerland

**DOI:** 10.1111/jiec.12486

**Published:** 2016-09-26

**Authors:** Florian Suter, Bernhard Steubing, Stefanie Hellweg

**Affiliations:** ETH Zurich, Institute of Environmental Engineering (IfU), John-von-Neumann-Weg 9, 8093 Zurich, Switzerland

**Keywords:** cascading, life cycle assessment (LCA), life cycle management (LCM), material flow analysis (MFA), substitution, wood resources

## Abstract

Sustainable use of wood may contribute to coping with energy and material resource challenges. The goal of this study is to increase knowledge of the environmental effects of wood use by analyzing the complete value chain of all wooden goods produced or consumed in Switzerland. We start from a material flow analysis of current wood use in Switzerland. Environmental impacts related to the material flows are evaluated using life cycle assessment–based environmental indicators. Regarding climate change, we find an overall average benefit of 0.5 tonnes carbon dioxide equivalent per cubic meter of wood used. High environmental benefits are often achieved when replacing conventional heat production and energy-consuming materials in construction and furniture. The environmental performance of wood is, however, highly dependent on its use and environmental indicators. To exploit the mitigation potential of wood, we recommend to (1) apply its use where there are high substitution benefits like the replacement of fossil fuels for energy or energy-intensive building materials, (2) take appropriate measures to minimize negative effects like particulate matter emissions, and (3) keep a systems perspective to weigh effects like substitution and cascading against each other in a comprehensive manner. The results can provide guidance for further in-depth studies and prospective analyses of wood-use scenarios.

**Supplementary Information:**

The online version of this article (doi:10.1111/jiec.12486) contains supplementary material, which is available to authorized users.

## Introduction

Wood can be a large sink for carbon dioxide (CO_2_) and is a multifunctional renewable resource suitable for various material and energy purposes. The sustainable use of wood can lower impacts on climate change and can decrease the nonrenewable resource demand. However, current developments in wood use challenge such advantages. Wood stocks are increasing in regions like the European Union (EU) (FAO 2010) and Switzerland (BAFU 2014) because of underused forests. As a result, the climate mitigation potential of forests is currently not exploited to the full extent. Such increasing wood stocks are less favorable for carbon sequestration than when they are utilized at their incremental growth rate (e.g., Walz et al. 2010; Werner et al. 2010). Additionally, the sequestration potential in aging forests or wood products is often much smaller than the mitigation potential of wood replacing nonwood products (UBA 2015). These developments call for new strategies facilitating sustainable wood mobilization and use. An important basis is the provisioning of a quantitative system analysis on environmental benefits and impacts.

So far, only a few studies go beyond the analysis of single environmental indicators (mainly climate-change impacts) or individual wood products and sectors (e.g., Werner et al. 2010; Windsperger et al. 2010; Palosuo et al. 2010). An enlargement of the system boundaries is, however, important in order to understand the wider implications of observed environmental effects (Gustavsson and Sathre 2010). Effects like material substitution (the replacement of a product by another one) or resource cascading (the quality-based sequential use of a resource) may especially influence large parts of a system. Resource cascading can improve the overall resource efficiency in general (e.g., Sirkin and ten Houten 1994; Haberl and Geissler 2000), but also for wood in particular (e.g., Dodoo et al. 2014; Sathre and Gustavsson 2006; Höglmeier et al. 2013). In the case of wood, environmental effects from cascading are often exceeded by those from the substitution of nonwood products (e.g., Sathre and Gustavsson 2006; Höglmeier et al. 2015).

The aim of this study is to assess the various environmental impacts and benefits associated with the wood value chain in Switzerland. Hereby, we include all wooden products produced or consumed in Switzerland and cover the whole value chain “from cradle to grave”. In order to do so, we (1) environmentally assess the individual components of the wood value chain, (2) assess the contribution of wood to the overall impacts of a product, and (3) estimate the potential environmental benefits from wood use when substituting other products. The results shall deliver guidance for further in-depth studies and prospective analyses to develop strategies for sustainable use of wood in the future.

## Methodology

In this study, a combination of material flow analysis (MFA) and life cycle assessment (LCA) allows the modeling of environmental impacts and benefits. Production data for wood products are based on annual statistics (BAFU 2012a; BFE 2012a, 2012a; ZPK 2011; BFS 2012) and reports (BAFU 2013; Lehner et al. 2014; BAFU and BFE 2014) of federal offices and industry associations. Environmental impact calculations are based on life cycle inventories (LCIs) from ecoinvent 3.1 in the cut-off allocation system model (Wernet et al. 2016).

### Material Flow Analysis and Life Cycle Assessment Model

In a first step, products were defined and product amounts calculated. The starting point was an existing MFA of wood use in Switzerland for the year 2011 (BAFU 2012a). This MFA provides a consistent view of the wood flows along the value chain. Flows are expressed in cubic meter (m^3^) solid wood equivalents. The high aggregation level, however, causes a loss of accuracy in product and process resolution compared to available statistics. In order to achieve an increased resolution in the model without losing too much consistency within this MFA, the statistical data have been allocated proportionally to the output quantities of the MFA processes. For example, statistical data for the production of different kinds of fiber boards and particle boards was summed up to the MFA process “board production”. In accord with the proportion of board production, each board type was allocated to the output amount of the respective MFA process. The resulting extended MFA provided the necessary level of detail for the environmental impact calculations. Product amounts were specified for domestically produced and consumed, as well as imported and exported products (table S6 in the supporting information available on the Journal's website). Product flows in the extended MFA were also expressed in m^3^ solid wood equivalents by using specific conversion factors for each product (table S5 in the supporting information on the Web).

In a second step, the environmental impacts associated with the product amounts were calculated. This included the following impact assessment methods: climate-change impact for a time horizon of 100 years (IPCC 2013); ReCiPe mid- and endpoints (Goedkoop et al. 2009); cumulative energy demand (CED) (Hischier et al. 2010); and ecological scarcity 2013 (Frischknecht and Büsser Knöpfel 2013).

**Figure 1:**
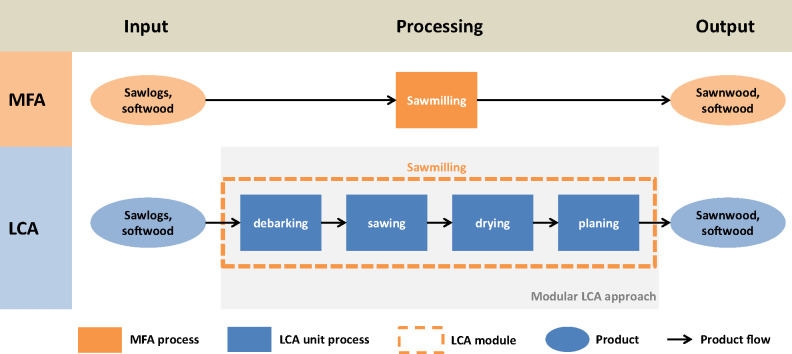
Schematic representation of the MFA and LCA combination on the simplified example of sawmilling. For the LCA part, all inventories (LCA unit processes) from the MFA process “sawmilling” were merged to an interconnected LCA module representing the sawmilling in the LCA. The product input (in this example, “sawlogs, softwood”) was cut off from the LCA module, and the production of this input is defined in another preceding LCA module. All interconnected modules of the value chain were then scaled with the product output amount of the appropriate MFA process (in this example, “sawnwood, softwood”). Product input amounts (in this example, “sawlogs, softwood”) were scaled according to the output amounts. LCA = life cycle assessment; MFA = material flow analysis.

LCIs on a unit process resolution were often not directly applicable to products of the extended MFA given that the resolution of inventory chains and MFA process chains differed from one another. A modular LCA approach (Steubing et al. 2016) was applied to represent the processes in the MFA supply chain through interconnected LCA modules. The LCA modules consist of a combination of predefined inventory chains from the ecoinvent 3.1 database and upstream cutoffs to delimit their system boundary (figure 1).

Environmental impacts of such a module consist of impacts from the represented process itself as well as impacts from all related upstream inputs except wood-based ones. Impacts from wood-based inputs are already accounted for in the modules producing these products. As such, this approach avoids a double counting of impacts.

Unlike the impacts of the domestically produced product amounts, impacts from imported and exported product amounts include also upstream impacts of wood processing. Domestic consumption impacts were calculated from domestic production impacts minus exported plus imported impacts. Some uncertainty in consumption impacts is caused from the diverging system boundaries in impact calculations for domestic production and trade.

The model representation of wood use in Switzerland consists of 52 processes that produce 40 wood-based products covering the different sectors of the wood market up to semifinished products. Besides energy, no products of the final processing step (e.g., houses, furniture) were included in the model because of nonexistent or inconsistent MFA and LCA data. Table S4 in the supporting information on the Web provides an overview of all products and processes used in the model.

### Short-Term Cascading Potential in Switzerland

The current annual net increment in Swiss forests of 7.4 cubic meters per hectare (m^3^/ha) exceeds the annually used amount of 6.6 m^3^/ha (BAFU 2015). Underused forests are mainly located in the mountainous region, where harvesting costs are higher than in the lowlands. The underuse suggests that an additional cascade use of wood would primarily lead to less wood extraction from the forest. That will not change as long as the demand of wood products does not increase substantially. To examine the potential benefits of an increased cascade in this situation, we quantified the primary wood-related impacts in products in comparison to total product impacts. This indicates the impact reduction that could be achieved by cascading. We call it here the “short-term cascading potential,” because the long-run demand for wood can be expected to increase because of additional environmental pressure (e.g., climate change). This long-term development would lead to substitutions of nonwood materials.

### Current Substitution Achievements in Switzerland

In order to assess current material and energy substitution effects from the use of wood products, a retrospective view was adopted. In Switzerland, most of the wood is utilized in energy (approximately 50%) and paper production (approximately 25%). The remaining share goes into the production of various commodities, namely, buildings (approximately 10%), furniture (approximately 3%), and packaging (approximately 3%) (BAFU 2012a; BAFU and BFH 2012; Lehner et al. 2014). For each of these services, production impacts from wood and nonwood alternatives were compared. Only paper was not considered, because substitution products are often a technological leap at the same time (Jeswani and Azapagic 2014) and non-wood-related substitution options were hardly available in the past. For each service (except energy), the point of substitution was at the level of semifinished products, that is, at the inputs to the service. In accord with the estimated demand (BAFU and BFH 2012; Lehner et al. 2014; BUWAL 2004), the wood products were distributed to each of the services and alternative nonwood products were defined (table 1). Benefits achieved by current wood use were calculated in three scenarios. One scenario reflects the likely benefits of a supposable substitution case for wood. A second scenario represents minimum benefits and a third the maximum benefits from wood use. The three scenarios differ in the composition of the substitution products to reflect variability and uncertainty in the assumptions.

**Table 1:** Assumed substitution between wood products and substitutes in the considered services

			Fraction of wood product in substitution scenario (%)		
Wood product	Substitute	Service	Minimum benefit	Likely benefit	Maximum benefit
Glued laminated timber	Steel, secondary	Construction	100	90	80
	Steel, primary	Construction	0	10	20
Sawnwood products	Concrete	Construction	0	39	70
	Brick	Construction	70	31	0
	Polyethylene	Packaging	10	9	0
	Aluminum, secondary	Packaging	0	0.9	8
	Aluminum, primary	Packaging	0	0.1	2
	Polypropylene	Furniture	20	10	0
	Steel, chromium, secondary	Furniture	0	9	16
	Steel, chromium, primary	Furniture	0	1	4
Fiberboard (hard and medium)	Gypsum fiberboard	Construction	70	70	70
	None	Furniture	30	30	30
Fiberboard (soft)	Rock wool	Construction	100	40	0
	Polystyrene	Construction	0	60	100
Particle board	Plaster	Construction	60	60	60
	Glass	Furniture	40	40	40
Plywood	None	Construction	70	70	70
	Steel, chromium, secondary	Furniture	30	27	24
	Steel, chromium, primary	Furniture	0	3	6
Heat from wood	Heat from natural gas	Heat	100	30	0
	Heat from light fuel oil	Heat	0	70	100
Electricity from wood	Electricity from mix CH	Electricity	100	100	100
Paper	None	Print	100	100	100

*Note*: For each of the three observed scenarios, the table gives the fraction of wood product involved in the substitution. Scenarios represent the degree of environmental substitution benefit for wood, going from minimum via likely to maximum benefits. Wood products without a direct substitute (labelled with ‘None’ in substitute column) are not considered for the calculations. CH = Switzerland.

Actual lifetimes of the products were expected to be equal for wooden materials and their substitutes given that these often depend on factors like overall product quality, fashion, and purchasing power of the consumer rather than on the material itself. Studies for furniture (e.g., Wenker 2012) confirm that such factors can play an important role for the disposal. Tables S8 and S9 in the supporting information on the Web provide further details to the selection of substitutes.

### End of Life

The calculation of substitution effects also includes the end-of-life (EoL) treatment. For each of the products, a treatment mix consisting of inert landfilling, recycling, and incineration was estimated (table 2), based on waste streams from the city of Zurich (Boucher 2014) and own assumptions. The cut-off allocation system of ecoinvent 3.1 already considers recycled content in the production of certain products. To avoid double counting, no further recycling benefits were given to these products. EoL effects of other products (in recycling and incineration) were captured with a system expansion. Landfilling received no credits. Possible losses from material collection were not considered. More details regarding EoL calculations are available in tables S10 and S11 in the supporting information on the Web.

**Table 2:** Share of each product going into the different end-of-life treatment options

		End-of-life treatment options and their shares					
	Commodity	Recycled content	Recycling		Incineration		Inert landfill
Type of products		Share (%)	Share (%)	System expansion	Share (%)	System expansion	Share (%)
Substituted products	Steel, secondary	100	0		0		0
	Steel primary	0	100	Steel, secondary	0		0
	Steel, chromium, secondary	100	0		0		0
	Steel, chromium, primary	0	100	Steel, chromium, secondary	0		0
	Aluminum, secondary	100	0		0		0
	Aluminum, primary	0	100	Aluminum, secondary	0		0
	Concrete	0	98	Gravel	0		2
	Polyethylene	0	5	Ethylene	95	Oil, natural gas, electricity mix CH	0
	Polypropylene	0	5	Propylene	95	Oil, natural gas, electricity mix CH	0
	Brick	0	100	Gravel	0		0
	Flat glass	0	50	Foam glass	0		50
	Gypsum fiberboard	0	35	Gypsum	0		65
	Rock wool	0	0		0		100
	Polystyrene	10	0		90	Oil, natural gas, electricity mix CH	0
	Plaster	0	0		0		100
Wood products	Glued laminated timber	0	0		100	Oil, natural gas, electricity mix CH	0
	Sawnwood products	0	0		100	Oil, natural gas, electricity mix CH	0
	Fiberboard (hard and medium)	10	0		90	Oil, natural gas, electricity mix CH	0
	Fiberboard (soft)	0	0		100	Oil, natural gas, electricity mix CH	0
	Particle board	20	0		80	Oil, natural gas, electricity mix CH	0
	Plywood	0	0		100	Oil, natural gas, electricity mix CH	0

*Note*: “Recycled content” represents the case were the recycling is already considered in the cut-off allocation system of ecoinvent 3.1. In the case of system expansion (recycling and incineration), replaced products are listed. CH = Switzerland.

In Switzerland, approximately 50% of the energetically used waste wood is combusted in municipal waste incineration with an electric efficiency of 15% and a thermal efficiency of 25%. The other 50% are combusted in plants for renewable waste with an electric efficiency of 15% and a thermal efficiency of 45%. All nonwood waste goes into municipal waste incineration.

The system expansion for end-of-life incineration includes electricity from the Swiss electricity mix (replaced by the produced end-of-life electricity) and heat from a mix of oil and natural gas (replaced by the produced end of life heat). The replaced heat mix varies between the scenarios and corresponds with the numbers given in table 1.

Difference of impacts from foam glass without and with glass cullet as input.

## Results

### Volume- and Impact-Based Perspective on Wood Use

In the subsequent section, impact shares of the wood products and processes are related to the total domestic consumption impacts, if not stated differently. Results are shown for ecological scarcity (eco scarcity 2013), ReCiPe total (ReCiPe, total), particulate matter formation (ReCiPe, PMFP), climate-change impacts (IPCC 2013), and cumulative energy demand for fossil energy (CED, fossil). Note that the climate-change impact of biogenic CO_2_ emissions are not included here, although for short-term effects they could be relevant in some applications, for example, direct wood use for heat production (Cherubini et al. 2016) (for a discussion, see section *Further Limitations* below). Abbreviations used in the subsequent parts are in the brackets. Results for further life cycle impact assessment methods and impact categories are available in figures S1 to S6 in the supporting information on the Web.

**Figure 2:**
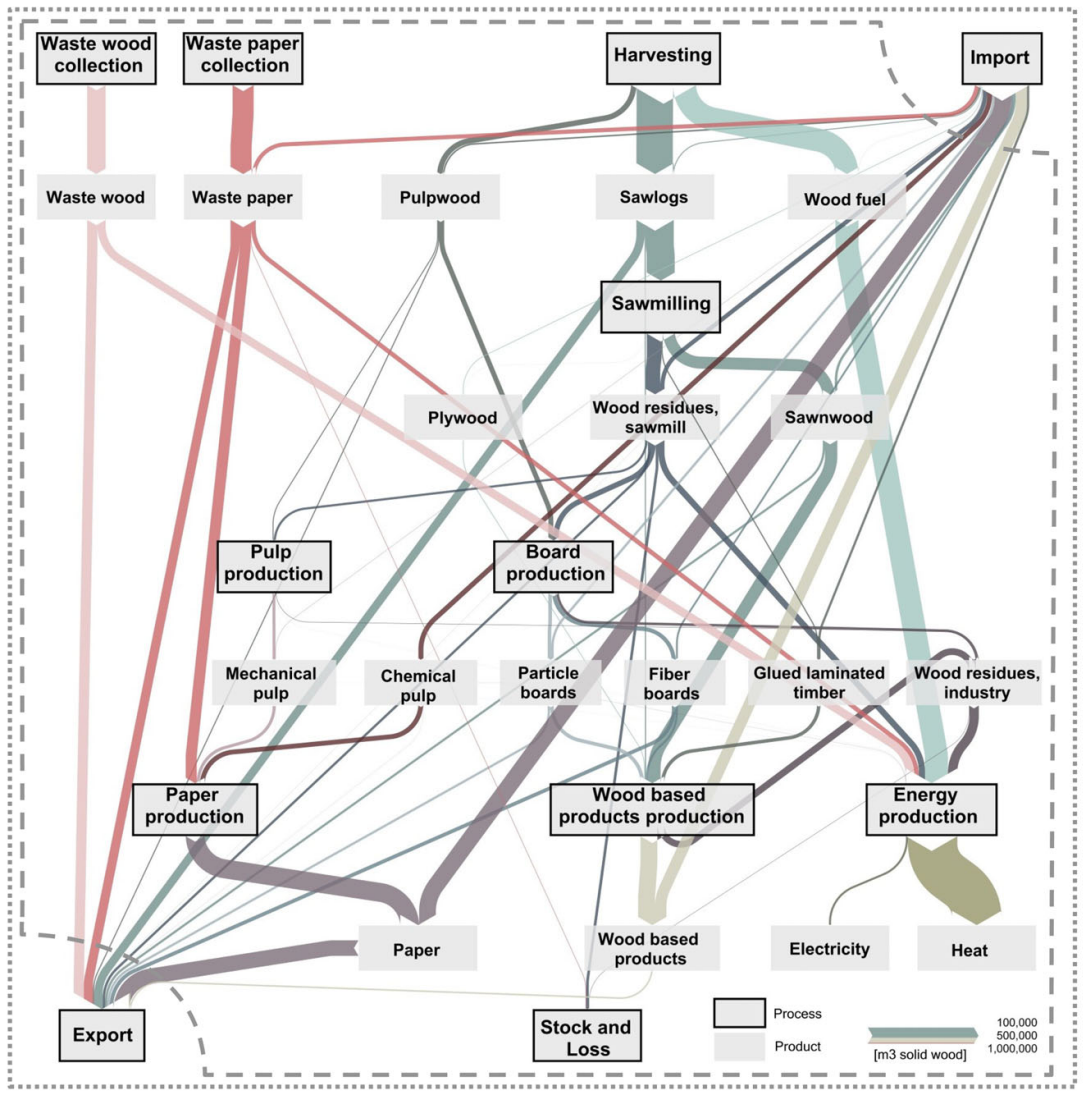
Wood flows in Switzerland for the year 2011. The thickness of arrows represents the wood volume of respective flows. The dashed line represents the system boundary for Switzerland, and the dotted line represents the overall system perspective, including trade. Although the production of wood-based products is shown here for the sake of completeness, it is only considered on the level of inputs (semifinished products like boards, glued laminated timber, and so on) in the model attributed to missing and incomplete LCA data. LCA = life cycle assessment.

Domestically, the largest amounts of wood in 2011 are processed within harvesting and energy production (approximately 6.5 and 5.3 million m^3^ wood, respectively) (figure 2). They are followed by sawmilling, collection of waste paper and waste wood, and paper production. Conversely, only rather small amounts of 0.8 and 0.2 million m^3^ of wood are processed in board and pulp production, respectively. Productwise, most of the wood is used for heat and paper, and a large part goes through sawmilling. Although produced and consumed amounts of wood match well, there is substantial trading of wood products with other countries. Most of the wood is exported in the form of paper, waste wood, waste paper, and sawnwood. Wood imports are concentrated in paper and products of the final processing step.

**Figure 3:**
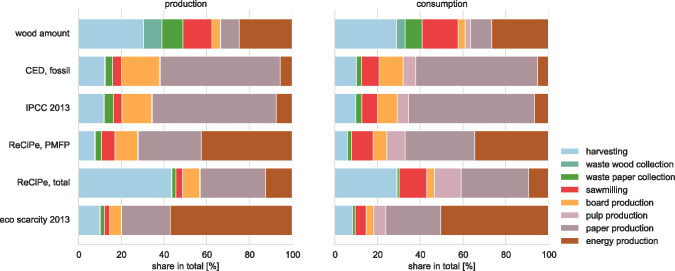
Comparison of process shares in the overall system based on volume (top) and impacts. The left-hand side represents the production perspective, the right-hand side the consumption one.

Shifting the perspective from a volume-based to an impact-based perspective dramatically changes the order of relevancy of products and processes (figure 3). Paper causes a large share of the overall consumption impacts (23% to 58% of overall production impacts according to the methods shown in figure 3). Other important contributors to overall impacts are energy production (6% to 57%) and board production (8% to 18%) as well as harvesting (8% to 44%).

Results show similarities for particulate matter formation and ecological scarcity. Particulate matter emissions mainly occur in combustion processes. Given that they are an important factor for the calculation of eco-points, they correlate with results from the ecological scarcity method. Results for climate-change impacts and fossil CED are also similar. Processes like board or paper production often have high fossil energy inputs. This causes high greenhouse gas (GHG) emissions, which explain the strong correlation. Processes that involve land use (e.g., harvesting) are attributed with high impacts in ReCiPe total because it weights land occupation heavily. The ReCiPe method, however, does not account for regional differences, neither in the intensity of forest management nor in biodiversity.

### Short-Term Cascading Potential

When impacts related to the primary wood in a product are compared to the total product's impact, two effects become apparent (figure S7 in the supporting information on the Web): First, primary wood shows very large impact shares with the ReCiPe total method. This could be, however, a model artefact from the heavy weighting of land occupation in this method not directly applicable for the case of Switzerland. Shares are also high with the ecological scarcity method, mainly attributed to the use of primary energy. At the same time, shares are generally significantly lowered when considering particulate matter and GHG emissions as well as cumulative fossil energy demand. Thus, only small benefits are achieved for these methods when wood cascading leads to a replacement of primary wood.

Second, in most products requiring higher levels of processing, the wood-material–related impacts are only secondary compared to impacts from other materials and process-related impacts. This decreases the environmental relevance of wood with increasing degree of processing.

### Impacts and Benefits of Current Wood Use

**Figure 4:**
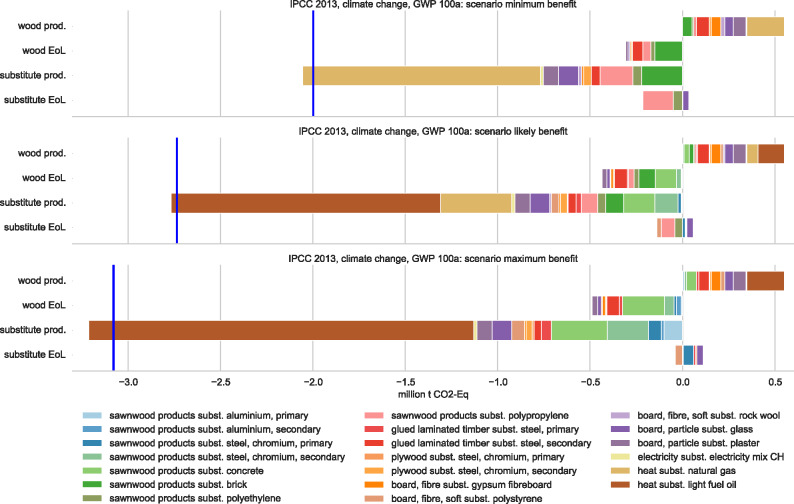
Substitution effects as climate change impacts (positive numbers) and benefits (negative numbers) from the production (prod., table 1) and end-of-life (EoL, table 2) treatment of current wood use in Switzerland. Production impacts of substitutes are negative numbers (given that they can be saved with the use of wood), whereas EoL benefits from substitutes are positive numbers (given that they are lost with the use of wood). Results are shown for the three substitution scenarios minimum (top), likely (middle), and maximum (bottom) benefit. For each scenario, a blue line indicates the cumulative total net effect from wood use.

In terms of GHG emissions, wood use is beneficial in most applications. Benefits are particularly high when replacing heat from oil or gas as well as energy-intensive products, such as primary metals, plastics, and concrete, in construction and furniture. In addition, notable environmental benefits are possible from EoL treatment. Especially for wood products, such benefits can be high given that most waste wood is incinerated directly. Such benefits can be even larger than production impacts. Figure 4 illustrates the substitution effects for production (prod.), EoL, and the total net effect (blue lines) for the three analyzed scenarios. The effects are shown from a wood perspective, meaning that production impacts for substitutes are negative (savings when wood is used instead) and EoL benefits positive (losses when wood is used instead).

Potential benefits from using wood-based materials vary considerably depending on impact method chosen and materials replaced. For instance, substitution benefits get much smaller when considering eco-points and particulate matter formation or even reversed, for example, in the case of land occupation. In such cases, wood products can have higher impacts then the use of nonwood alternatives. Results for these methods are available in figures S8 to S11 in the supporting information on the Web.

### Displacement Factors for Greenhouse Gases

Substitution effects across the substitution options considered result in an average benefit of 0.5 tonnes carbon dioxide equivalent per cubic meter (t CO_2_-eq/m^3^) of wood used (table 3). Of these, an average benefit of 0.3 and 0.4 t CO_2_-eq/m^3^ of wood comes from material options only and energy options only, respectively. In addition, an average benefit of 0.2 t CO_2_-eq/m^3^ wood used results from the EoL treatment of wood. The much lower benefit from waste wood incineration compared to primary wood incineration is the result of a relatively low thermal efficiency in Swiss municipal waste incineration.

**Table 3:** Average reduction in climate change impacts per cubic meter of wood invested for the three different substitution scenarios

	Benefit per scenario (t CO_2_-eq/m^3^ wood)			
	Minimum benefit	Likely benefit	Maximum benefit	Average across scenarios
Material use				
Material substitution	0.18	0.25	0.34	0.26
End-of-life substitution	0.22	0.23	0.19	0.21
Energy use				
Energy substitution	0.31	0.47	0.54	0.44
Overall substitution	0.35	0.47	0.53	0.45

*Note*: Numbers are given for benefits from material substitution, end-of-life substitution, energy substitution, and overall substitution. The average benefit represents the average over all three scenarios. Numbers are rounded.

Substitution factors are the weighted average over all material based substitutions (without end of life).

Substitution factors are the differences from material substitution with and without end of life included.

Substitution factors are the weighted average over all energy based substitutions (excluding waste wood, waste paper, and energy production in production processes itself).

t CO_2_-eq/m^3^ = tonnes carbon dioxide equivalent per cubic meter.

Generally, the direct substitution effect is higher when wood is used energetically. A material use followed by an energetic EoL utilization is, however, even more preferable (provided that the waste wood is combustible and losses are minimal). The results show, again, a strong dependency on the substitution assumptions made (differences between different substitution scenarios). Table S3 in the supporting information on the Web provides the displacement factors on a product basis.

### Relevance of Cascading and Substitution

**Figure 5:**
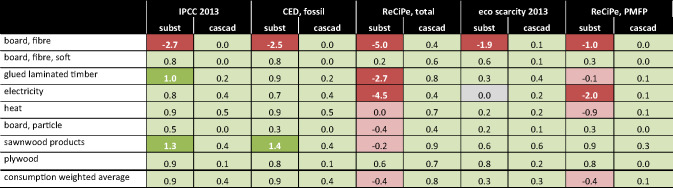
Factors indicating the environmental performance of nonwood substitution (subst) and wood-wood substitution (short-term cascading, cascad) for the different products and impact methods. Positive factors indicate decreasing impacts, negative factors increasing impacts. Factors indicate how much better (positive numbers with green background) or worse (negative numbers with red background) nonwood and wood-wood substitution perform to the reference case without substitution in terms of environmental impacts. Numbers are rounded. Reading example: For the production of plywood, climate-change impacts are 90% lower as when comparable products from other materials would be produced (substitution). However, climate-change impacts are only reduced by 10% when waste wood replaces primary wood in plywood production (short-term cascading).

Factors in figure 5 indicate how much impacts are reduced (positive numbers) or increased (negative numbers) with a substitution (including EoL) or short-term cascading pattern in comparison to the reference without such patterns. In the case of substitution, the difference is between the environmental impacts from substitute and wood product. The factor is the ratio of this difference to the impact of the substitute product. In the case of short-term cascading, the difference is between the environmental impacts from the wood product with and without primary wood included. The factor is the ratio of this difference to the impacts of the wood product, including primary wood. Negative factors result from impacts higher than as in the reference pattern. Factors greater are the result of wood product impacts below zero, that is, benefits from EoL treatment are greater than production impacts.

Regarding GHG emissions and fossil fuel demand, environmental savings are higher for effects from wood substitution than from short-term wood cascading. The opposite is the case when considering land occupation or particulate matter formation. Overall, results indicate that wood should be used as efficient as possible (positive cascading effects) and, at the same time, preferably should replace nonwood products (positive substitution effects). Results, however, also show a large variation between environmental effects of different products. In the case of fiberboard, substitution performs very poorly given that gypsum fiberboard as an alternative has lower environmental impacts.

## Discussion

### Environmental Impacts from Wood Use

The analysis of the wood value chain in Switzerland shows different environmental patterns.

First, wood supply from harvesting causes comparably small environmental impacts, particularly in terms of GHG emissions and fossil energy demand. ReCiPe total weights land occupation heavily and attributes land-intensive processes (e.g., harvesting) with high impacts for biodiversity loss. The ReCiPe method does, however, not distinguish between regional differences, neither in the intensity of forest management nor in biodiversity. Such high impacts concern mainly intensively oriented harvesting and management systems with high effects on biodiversity like clear-cuts and plantations. More extensively oriented harvesting systems, such as selective harvesting, have almost no effect on the overall biodiversity (Chaudhary et al. 2016). In the case of Switzerland, there is a legal obligation to use forests sustainably, and only selective harvesting is applied. Thus, ReCiPe total most likely overestimates impacts in the context of this study.

Second, wood use in energy provisioning results in small GHG emissions as well. At the same time, the combustion process leads to high particulate matter emissions. This is a known issue (especially in small-scaled heating facilities) (Szidat et al. 2007), also discussed in federal administrations (e.g., BAFU 2012). Solutions (like particle filters) are available, but still not implemented in all small-scale heating systems.

Third, each m^3^ of wood in the production of wood-based boards causes comparatively high environmental impacts with nearly all impact methods. The most important contributors are chemicals, resins, and the process energy required. However, the amount of boards is rather small (figure 2), which leads to small overall impacts in Swiss consumption.

Fourth, wood in the pulp and paper sector triggers the highest impacts within many categories. This reflects the particularly high energy consumption of this sector, also found in other studies (e.g., Silva et al. 2015). Paper producers are aware of this issue and try to reduce such impacts, for example, by replacing fossil fuels as energy carrier by biomass-based energy mainly incurred during the production itself (CEPI 2013). A further source of impacts is the use of chemical agents in the production. Impacts would be, however, even higher without the advanced recycling scheme. For Switzerland, 73% of the pulp consumed in paper production is from recycled paper (ZPK 2014). Additionally, the use of graphic paper will probably decline in the future because of an increasing use of electronic media.

Fifth, environmental impacts from wood use in Switzerland are strongly influenced by cross-boundary wood trade. Hence, the production abroad plays an important role for consumption impacts in Switzerland. This result is in line with other studies (e.g., Werner et al. 2010). Differences in impacts related to specific production conditions in Switzerland and abroad are, however, not represented sufficiently in the results because LCIs used in this study are not regionalized. For example, biodiversity impacts are very sensitive to the particular location (Chaudhary et al. 2015).

The modules representing MFA processes are output oriented. As a consequence, each module matches with the wood-based output quantity of the underlying process (the functional unit of the module), but not necessarily with the wood-based input quantities. This leads to the trade-off that inventory chains do not have to match exactly with material flows anymore.

Additional impacts can occur during the use phase of a product, which has not been considered in this work. Indoor use especially may lead to an increased exposure to emitted volatile organic compounds from adhesives and wood itself. If products are not managed accordingly, this can lead to health effects (Chaudhary and Hellweg 2014).

### Short-Term Cascading Effects

A burden-free supply of waste wood was assumed for the short-term cascading potentials, that is, impacts occurring from collection and sorting of waste wood were neglected. At the same time, potential process adjustments to handle waste wood were neglected. Further, harvesting is not necessarily the point of substitution between primary and waste wood. A shift further downstream in the production chain could lead to additional savings, for example, from a reduced or removed energy demand to dry the provided wood. However, Höglmeier and colleagues (2014) show that savings in process energy from a reduced drying effort for waste wood are only small. They conclude that process adjustments have only a minor influence on potential cascading advantages. Thus, the selected approach allows for a consistent assessment of short-term cascading potentials.

In terms of GHG emissions and cumulative fossil energy demand, results show a limited short-term cascading potential. As in the example of wood boards (González-García et al. 2009), production-based impacts are mainly affected by ingredients like chemicals or process energy. In contrast to these low potentials, short-term cascading can lead to rather good results when focusing on ReCiPe total or ecological scarcity. This refers, however, mainly to the impacts of land occupation, which are not regionally distinguished in these methods. As shown in a recent study (Chaudhary et al. 2016), many selective harvesting systems, as practiced in Switzerland, have a negligible or small impact. Therefore, land-use–related impacts are probably overestimated by ReCiPe.

Höglmeier and colleagues (2014) show that a cascade use of wood soon loses its advantages entirely with declining collection efficiency for waste wood given that this hinders or prohibits an efficient use for energy. Thus, attention should be paid to the avoidance of losses as well as to an effective energy-based EoL treatment. Further, an extended cascading of wood (in the sense of using more waste wood in material-related applications) should not lead to increasing primary wood amounts going directly into energy production. Otherwise, the result would be a replacement of waste wood (lower-quality wood) with primary wood (higher-quality wood) in energy production. From a resource point of view, it is currently preferable to use high-quality wood in materials.

Despite these results for short-term cascading effects, wood cascading is gaining in importance for the case when wood becomes a scarce resource in the future. Its potential to enable additional material and energy services by allowing a more intense use of limited biomass resources (Dodoo et al. 2014) can strongly support substitution efforts under such circumstances.

### Substitution Effects and Carbon Displacement Factors

Substitution with renewables often leads to a decrease in the consumption of nonrenewables and this can greatly contribute to reducing certain environmental impacts (Gustavsson and Sathre 2010). In the case of cumulative fossil energy demand and GHG emissions, different studies acknowledge such substitution benefits, in particular, also for wood. Sathre and O'Connor (2010) did a meta-analysis on such studies, analyzing GHG displacement factors of wood product substitutions. They found an emission reduction of roughly 3.9 t CO_2_-eq per metric tonne of dry wood used. Assuming a density of 500 kilograms per m^3^, this corresponds to an approximate reduction of 2 t CO_2_-eq/m^3^ of wood. A study about wood use in Switzerland found a reduction potential of 1.3 t CO_2_-eq/m^3^ of wood used (Werner et al. 2010). The calculated average reduction of 0.5 t CO_2_-eq/m^3^ of wood used is comparably low. The results of the different studies are, however, hardly comparable because of differences in system boundaries and basic assumptions like the handling of product life spans or the selection of studied products. Still, all studies show a clear overall GHG benefit from using wood instead of an equivalent amount of alternative materials. Wood products often have a much smaller demand for fossil energy in the production.

The consideration of other impact categories leads to more-diverse substitution effects, which are often less in favor of wood. In energy supply, this can lower major GHG benefits significantly (Steubing 2013). In material use, benefits fluctuate strongly with the chosen impact method, for example, in construction (Heeren et al. 2015). Wood often loses its benefits when it comes to air emissions from combustion (like particulate matter emissions) or land-use impacts (especially in unsustainably managed forests). Wood and wood flows have to be managed accordingly to maximize environmental advantages.

### Environmental Potentials under Scarce Resources

The assessment of the short-term wood cascading performance was done under the assumption that wood availability from forests is larger than wood demand, as is currently the case in Switzerland (BAFU 2015). Thereby, cascading would primarily lead to even less wood extraction from the forest, unless demand for wood products increases. In such a scenario, cascading hardly affects GHG emissions and fossil energy demand reduction. However, under a shortage of primary wood, cascading would ensure the supply of wood that could substitute other less environmentally favorable materials, leading to benefits in terms of GHG emissions and fossil energy demand. Table 3 provides an approximation for such a “long-term cascading performance” in consideration of GHG emissions. Under the assumption of no material losses, a material use followed by an energy use can be preferable over a direct energy use. Wood cascading is, however, always a material downcycling. Thus, an extended combination of substitution and cascading can only further improve the environmental performance of wood use, if the losses are kept small and a final energy utilization of the wood is not affected.

### Further Limitations

The focus on the status quo does not account for future effects. Yet, wood can have a long use phase (e.g., in buildings) whereby certain environmental effects arise only in the future. Such environmental impacts may be different to the ones occurring today. For example, a delay in the energetic utilization of wood may substitute cleaner future energy sources and can lead to less-favorable benefits as when used today (Gärtner et al. 2013). The focus on the status quo does also not account for carbon storage effects in wood products. Overall, such storage effects are less significant than substitution effects from wood products avoiding fossil emissions (Sathre and O'Connor 2010).

Environmental impacts of most end products have been approximated by impacts of semifinished products in the analysis. This approach may challenge a comparison on a functional level recommended by Gustavsson and Sathre (2010). Main impact sources should, however, still be captured, given that the final process steps often only contribute to a minor fraction of the overall impacts (e.g., for furniture) (Wenker and Rüter 2012). Often, main hotspots are the provisioning of resources or components and not so much the final combination of those (e.g., Sathre and González-García 2014; Iritani et al. 2014).

The omission of climate-change impacts of biogenic CO_2_ emissions (Cherubini et al. 2011, 2012; Levasseur et al. 2013) makes the direct use of wood as energy source appear better than it is, in terms of climate-change impacts (Cherubini et al. 2016). Further, the quantification of cascading and substitution effects can involve long time spans where effects such as biogenic carbon sequestration could become influential (leading to an additional benefit for material uses of wood in long-term applications, e.g., building construction). For the assessment of biogenic carbon, the temporal distribution of emissions is relevant and needs to be modeled, which was beyond the scope of the current study. The effect of biogenic CO_2_ emissions depends strongly on the time horizon considered: Whereas their impact is considerable in time frames of 100 years or less (Cherubini et al. 2011), the long-term effects are negligible in comparison to fossil CO_2_. Therefore, with regard to the long-term climate effects, our results are rather robust, whereas the short-term climate effects of wood could change significantly and might, in some applications, even challenge the benefits of using wood over alternative materials or energy sources, if biogenic carbon emissions were considered (Steubing 2013; Johnson and Tschudi 2012). A follow-up publication, modeling the temporal pattern of wood use and analyzing effects from biogenic carbon emissions on (short-term) climate impacts from wood use in Switzerland, is in preparation.

## Conclusions

This study highlights the environmental performance of wood for various impact categories using Switzerland as an example. It shows that the use of wood contributes to mitigate environmental impacts, for example, in terms of GHG emissions and fossil energy demand reduction. Main savings are achieved through substitution of other materials and energy. Nevertheless, wood also has its environmental limitations. Therefore, a deliberate and well-considered use of wood is required to maximize its environmental advantages while concurrently reducing its disadvantages. In particular, attention should be paid to the following points:

- Environmental hotspots in wood application, like the potentially high particulate matter emissions during combustion or effects on land occupation and biodiversity from harvesting, should be addressed (particle filter and sourcing of wood from forests with selection harvesting management) to minimize negative effects.
- A cascade-like use of wood can help to use the resource more efficiently. However, from an environmental point of view, it is particularly relevant which other products are substituted during the cascade and how completely and efficiently wood can be used for energy production in the end. Additionally, it is more important to increase the use of primary wood under the current underuse of the resource.
- A systems perspective, including the complete value chains of all wooden products and their substitutes, allows for a weighting of impacts from different use options against one another. This can help to optimize the wood use in terms of minimizing system-wide environmental impacts.

## Supplementary Information

Supporting info item
