# Supplementary material for: Life Cycle Impacts and Benefits of Wood along the Value Chain: The Case of Switzerland
Source: J Ind Ecol. 2016 Sep 26;21(4):874–86. doi: 10.1111/jiec.12486 (PMC13061803; doi:10.1111/jiec.12486)
Supplement: Supplementary file 1 — Supporting info item [file 44498_2017_2104006_MOESM1_ESM.pdf]

SUPPORTING INFORMATION FOR:

Suter, F., B. Steubing, and S. Hellweg. 2016. Life cycle impacts and benefits of wood along the value chain: The case of Switzerland. *Journal of Industrial Ecology.*

Summary

This supporting information provides additional information on the model inputs, model structure, assumptions, and LCIA results for further impact methods.

Contents

Impact methods S-3
Basic assumptions S-4
Additional figures and results S-5
    Wood amounts S-5
        Wood amounts from a process perspective S-5
        Wood amounts from a product perspective S-6
    Absolute environmental impacts of wood use S-7
        Impacts from a process perspective S-7
        Impacts from a product perspective S-8
    Relative environmental impacts of wood use S-9
        Impact shares from a process perspective S-9
        Impact shares from a product perspective S-10
    Impact share of primary wood S-11
    Substitution effects S-12
    Displacement factors for greenhouse gases S-15
Data basis S-16
    Wood use S-16
        Modelled processes and products with clustering and ecoinvent activities S-16
        Units and conversion factors S-21
        Amounts S-25
    Conversion of energy to wood amounts S-29
    Substitution S-30
        Substitution products, factors and ecoinvent activities S-30
        Substitution parameters S-32
    End of Life S-33
        End of Life parameters S-33
        End of Life ecoinvent activities S-35
References S-37

Impact methods

Table S1: Impact methods used with respective units and abbreviations.

Method	Unit	Abbreviation
IPCC 2013, climate change, GWP 100a	kg CO_2-Eq	IPCC 2013
ReCiPe Endpoint (H,A), total, total	points	ReCiPe, total
ReCiPe Endpoint (H,A), human health, total	points	ReCiPe, hum
ReCiPe Endpoint (H,A), resources, total	points	ReCiPe, res
ReCiPe Midpoint (H), marine eutrophication, MEP	kg N-Eq	ReCiPe, MEP
ReCiPe Midpoint (H), metal depletion, MDP	kg Fe-Eq	ReCiPe, MDP
ReCiPe Midpoint (H), natural land transformation, NLTP	m2	ReCiPe, NLTP
ReCiPe Midpoint (H), urban land occupation, ULOP	m2a	ReCiPe, ULOP
ReCiPe Midpoint (H), particulate matter formation, PMFP	kg PM10-Eq	ReCiPe, PMFP
ReCiPe Midpoint (H), agricultural land occupation, ALOP	m2a	ReCiPe, ALOP
ReCiPe Midpoint (H), freshwater ecotoxicity, FETPinf	kg 1,4-DCB-Eq	ReCiPe, FETPinf
ReCiPe Midpoint (H), climate change, GWP100	kg CO2-Eq	ReCiPe, GWP100
ReCiPe Midpoint (H), terrestrial acidification, TAP100	kg SO2-Eq	ReCiPe, TAP100
ReCiPe Midpoint (H), ionising radiation, IRP_HE	kg U235-Eq	ReCiPe, IRP_HE
ReCiPe Midpoint (H), freshwater eutrophication, FEP	kg P-Eq	ReCiPe, FEP
ReCiPe Midpoint (H), human toxicity, HTPinf	kg 1,4-DCB-Eq	ReCiPe, HTPinf
ReCiPe Midpoint (H), water depletion, WDP	m3	ReCiPe, WDP
ReCiPe Midpoint (H), fossil depletion, FDP	kg oil-Eq	ReCiPe, FDP
ReCiPe Midpoint (H), marine ecotoxicity, METPinf	kg 1,4-DCB-Eq	ReCiPe, METPinf
ReCiPe Midpoint (H), photochemical oxidant formation, POFP	kg NMVOC	ReCiPe, POFP
ReCiPe Midpoint (H), terrestrial ecotoxicity, TETPinf	kg 1,4-DCB-Eq	ReCiPe, TETPinf
ReCiPe Midpoint (H), ozone depletion, ODPinf	kg CFC-11-Eq	ReCiPe, ODPinf
ecological scarcity 2013, total, total	UBP	eco scarcity 2013
cumulative energy demand, fossil, non-renewable energy resources, fossil	MJ-Eq	CED, fossil
cumulative energy demand, wind, renewable energy resources, kinetic (in wind), converted	MJ-Eq	CED, wind
cumulative energy demand, nuclear, non-renewable energy resources, nuclear	MJ-Eq	CED, nuclear
cumulative energy demand, primary forest, non-renewable energy resources, primary forest	MJ-Eq	CED, forest
cumulative energy demand, geothermal, renewable energy resources, geothermal, converted	MJ-Eq	CED, geo
cumulative energy demand, water, renewable energy resources, potential (in barrage water), converted	MJ-Eq	CED, water
cumulative energy demand, solar, renewable energy resources, solar, converted	MJ-Eq	CED, solar
cumulative energy demand, biomass, renewable energy resources, biomass	MJ-Eq	CED, biomass

Basic assumptions

Table S2: Basic assumptions taken for calculations in this study.

Subject	Assumption	Basis
Lifetimes	Lifetimes are the same between substituted products. It is assumed that actual lifetimes of commodities depend more on factors like overall product quality, fashion and financial freedom of the consumer than on the used material itself.	
Waste wood incineration	All energetically used waste wood is incinerated in combustors for renewable resources and municipal waste incineration plants (BFE categories 19 and 20). It is divided equally on this to facilities. No waste wood is burned in woodworking workshops.	BFE (2012): Schweizerische Holzenergiestatistik: Erhebung für das Jahr 2011
Wood residues incineration	Wood automatic firing facilities within woodworking workshops (BFE categories 13, 15 and 17) burn only internally produced wood residues. Approximately 65% of the totally energetically used wood residues are burned in such facilities. The resulting energy corresponds to approximately 28% of the totally produced energy with automatic firing facilities. For environmental impact calculations this percentage has to be subtracted from the total amount of energy produced with such facilities. Otherwise, calculations lead to a double counting of produced energy from wood residues since the internally produced energy from wood residues is already included in ecoinvent processes.	BFE (2012): Schweizerische Holzenergiestatistik: Erhebung für das Jahr 2011
Incineration of non-wood products	When incinerated, all non-wood based products are burned only in municipal waste incineration plants.	
Collection losses	Collection losses in the End of Life treatment are not considered.	

Additional figures and results

Wood amounts

The subsequent part shows the wood amounts in Switzerland (based on the year 2011) from a process and product perspective, respectively. Numbers are shown in absolute as well as relative terms, and subdivided into domestic production and consumption as well as import and export.

Wood amounts from a process perspective

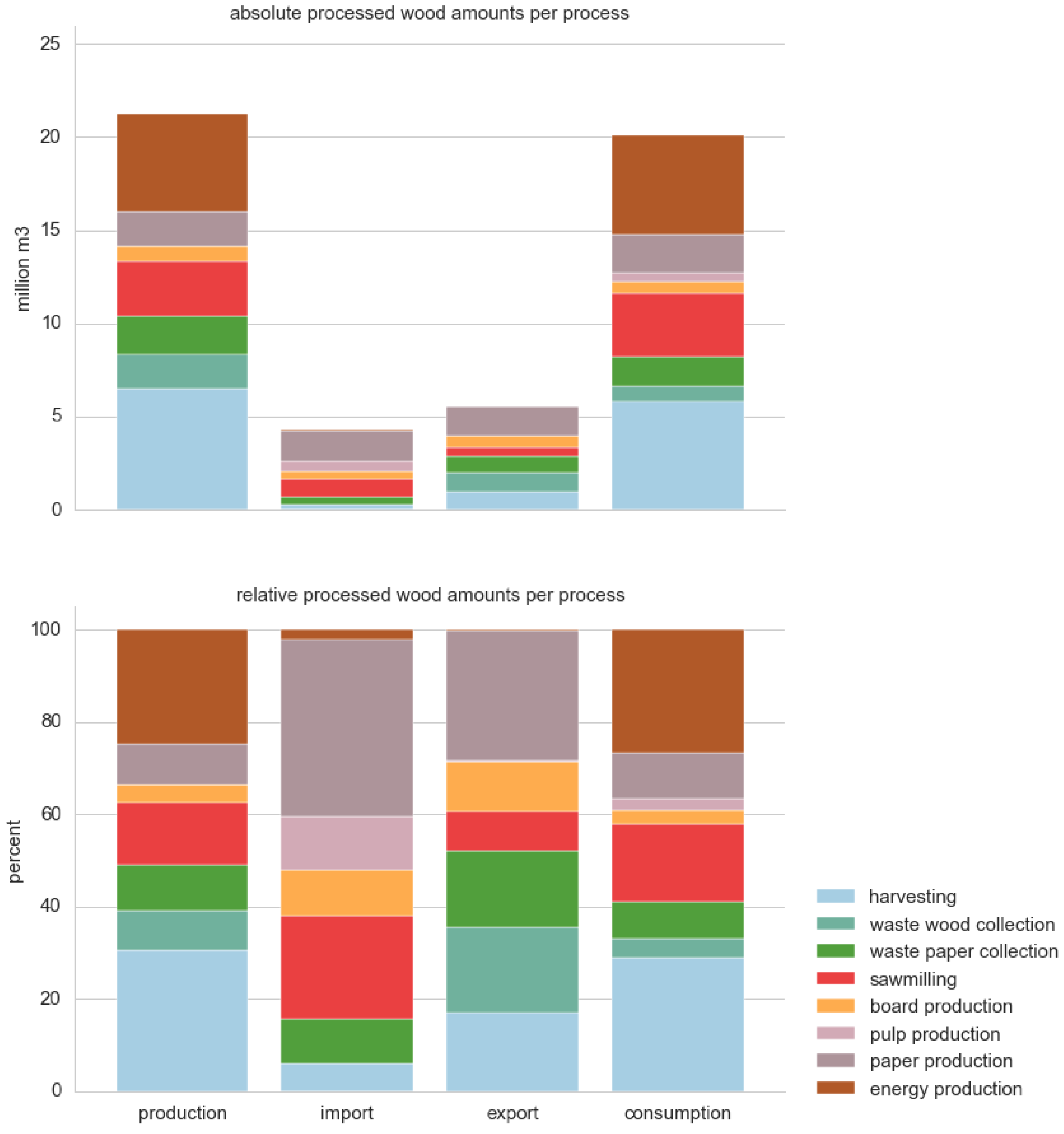

Figure S1: Absolute (top) and relative (bottom) wood amounts processed in the different processes for the year 2011.

Wood amounts from a product perspective

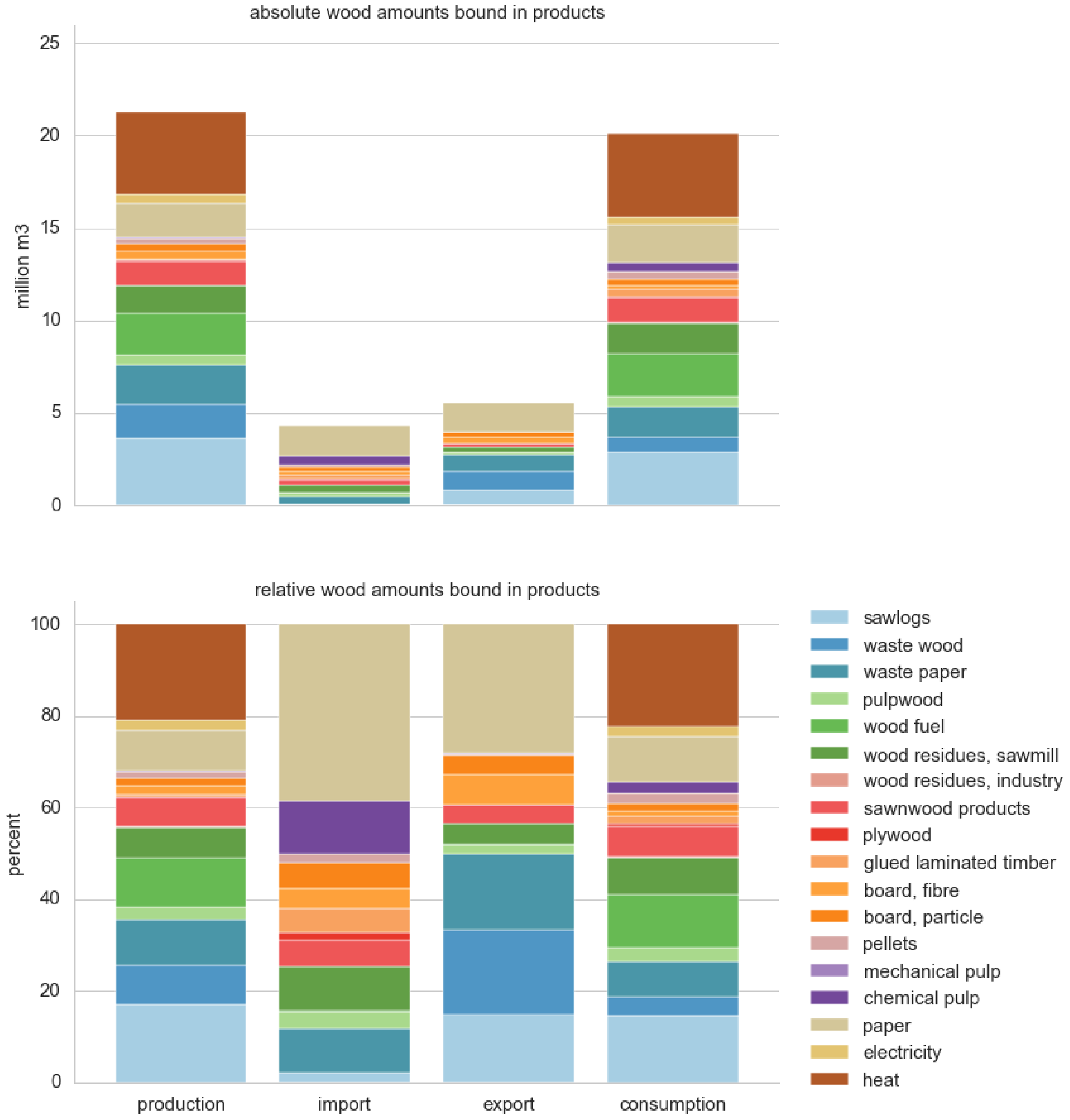

Figure S2: Absolute (top) and relative (bottom) wood amounts contained in products for the year 2011.

Absolute environmental impacts of wood use

The subsequent part shows different environmental impacts of wood use in Switzerland from a process and product perspective, respectively. Numbers are subdivided into domestic production and consumption as well as import and export.

Impacts from a process perspective

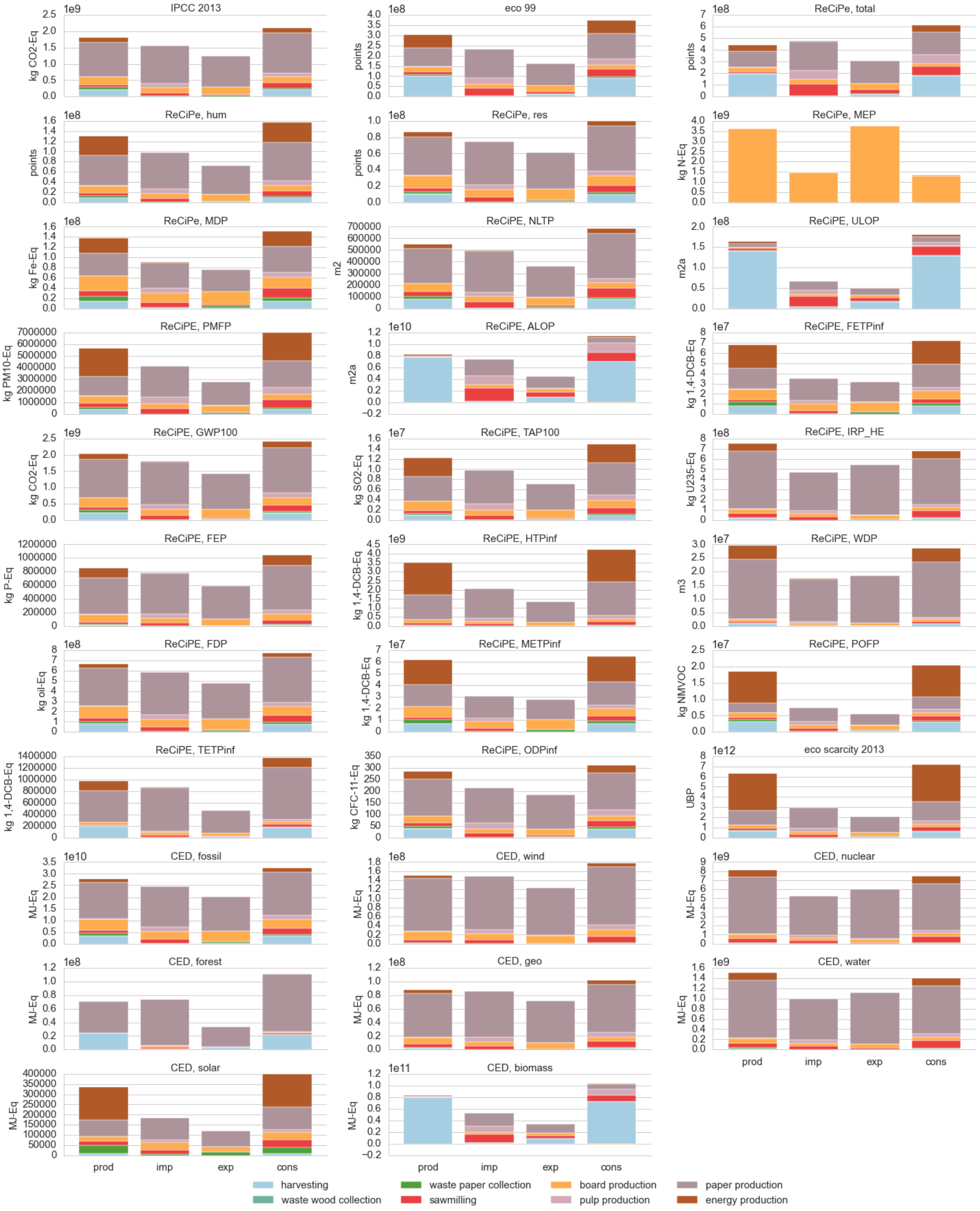

Figure S3: Environmental impacts of domestic production and consumption as well as import and export of wood in different processes for the case of Switzerland. Results are shown for various impact methods. Negative numbers in consumption are the result of the deviating impact calculation for the production (only includes respective process-level without upstream processes) and import/export (includes entire life cycle).

Impacts from a product perspective

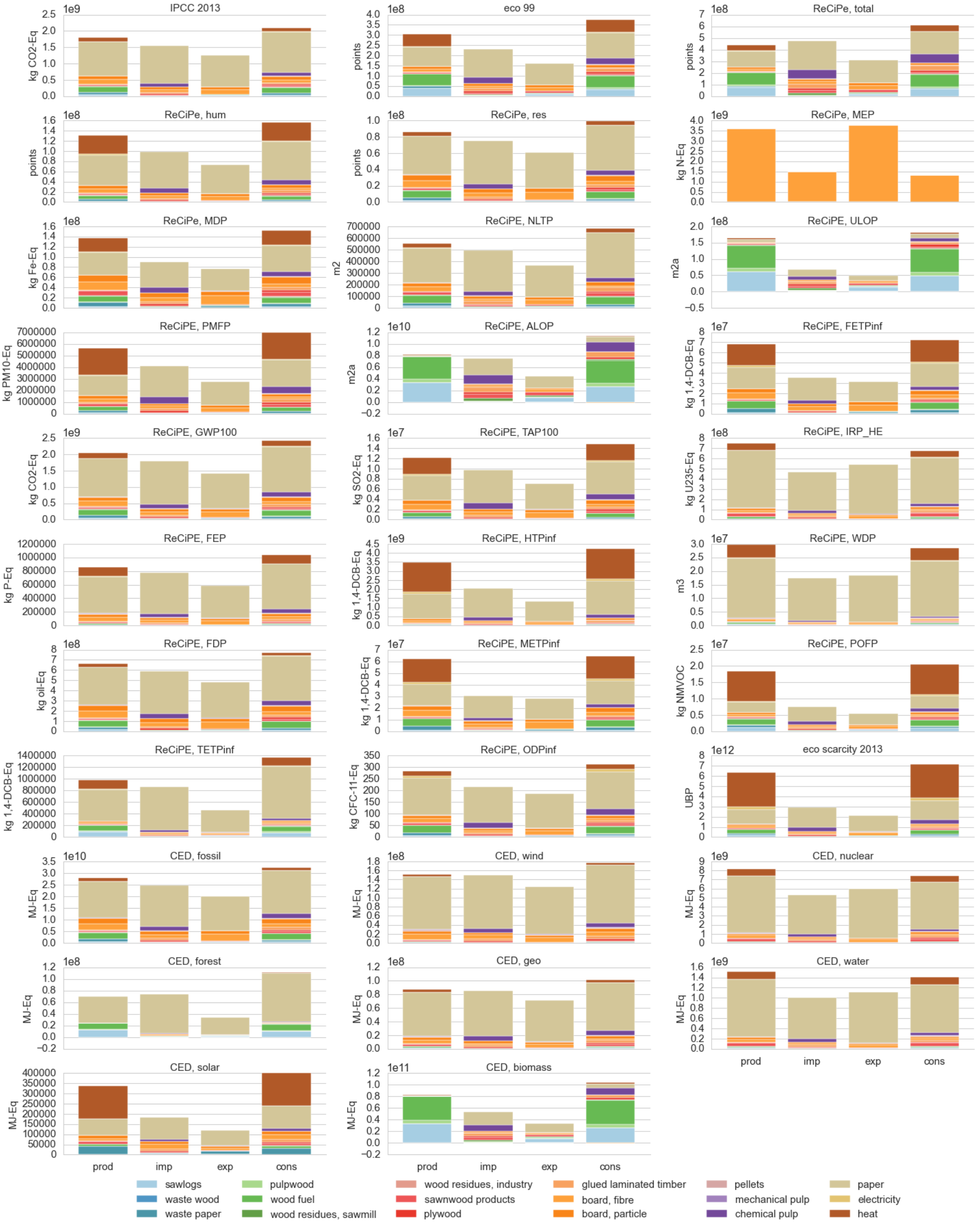

Figure S4: Environmental impacts of domestic production and consumption as well as import and export of wood in different products for the case of Switzerland. Results are shown for different impact methods. Negative numbers in consumption are the result of the deviating impact calculation for the production (only includes respective process-level without upstream processes) and import/export (includes entire life cycle).

Relative environmental impacts of wood use

The subsequent part shows environmental impact shares from a process and a product perspective.

Impact shares from a process perspective

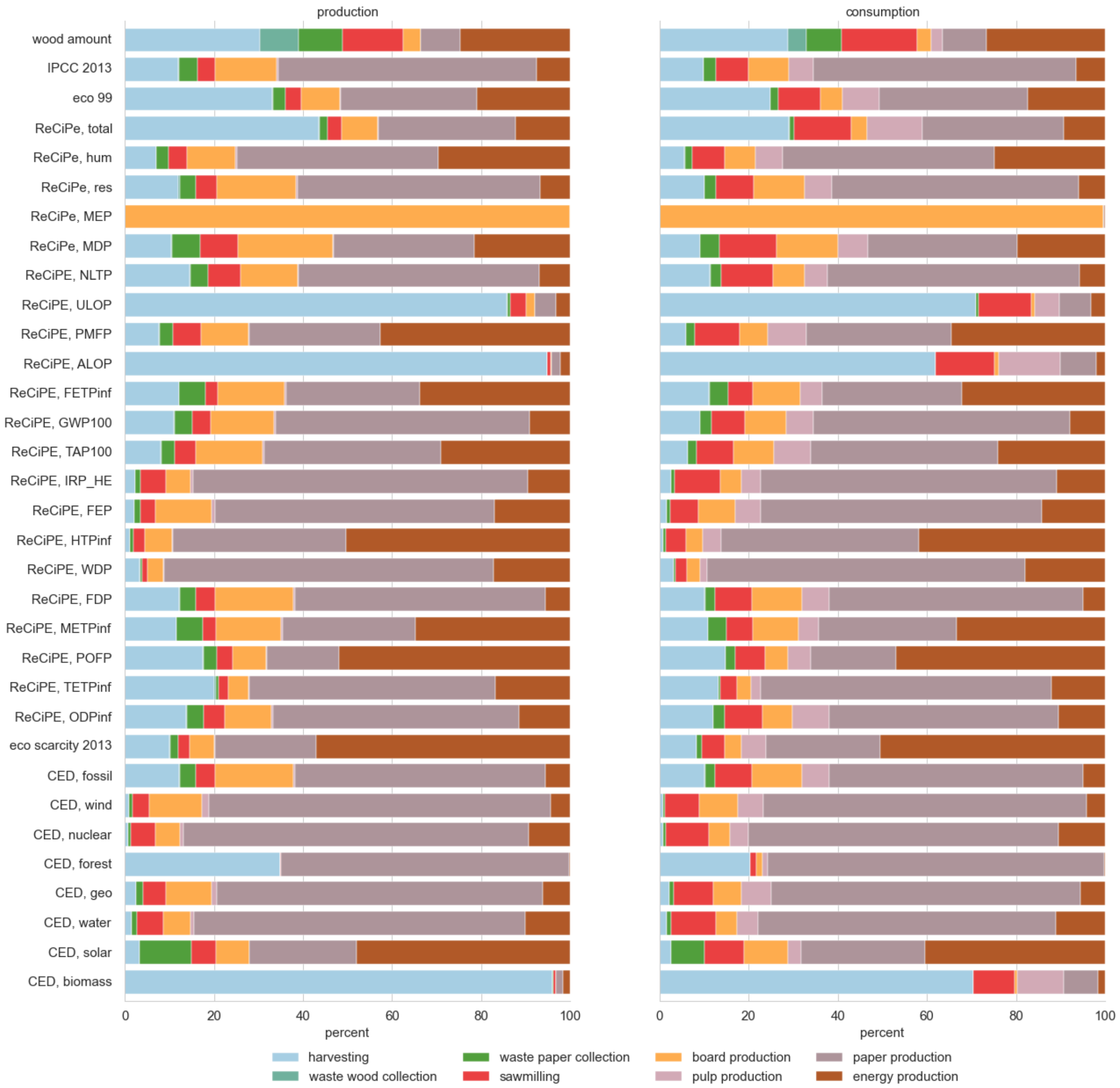

Figure S5: Impact share of the produced (left side) and consumed (right side) amount of wood in the different processes. The impact shares are shown for different methods. Additionally, shares for the produced and consumed amount of wood in the different processes are shown in the bar at the top of the figures (wood amount).

Impact shares from a product perspective

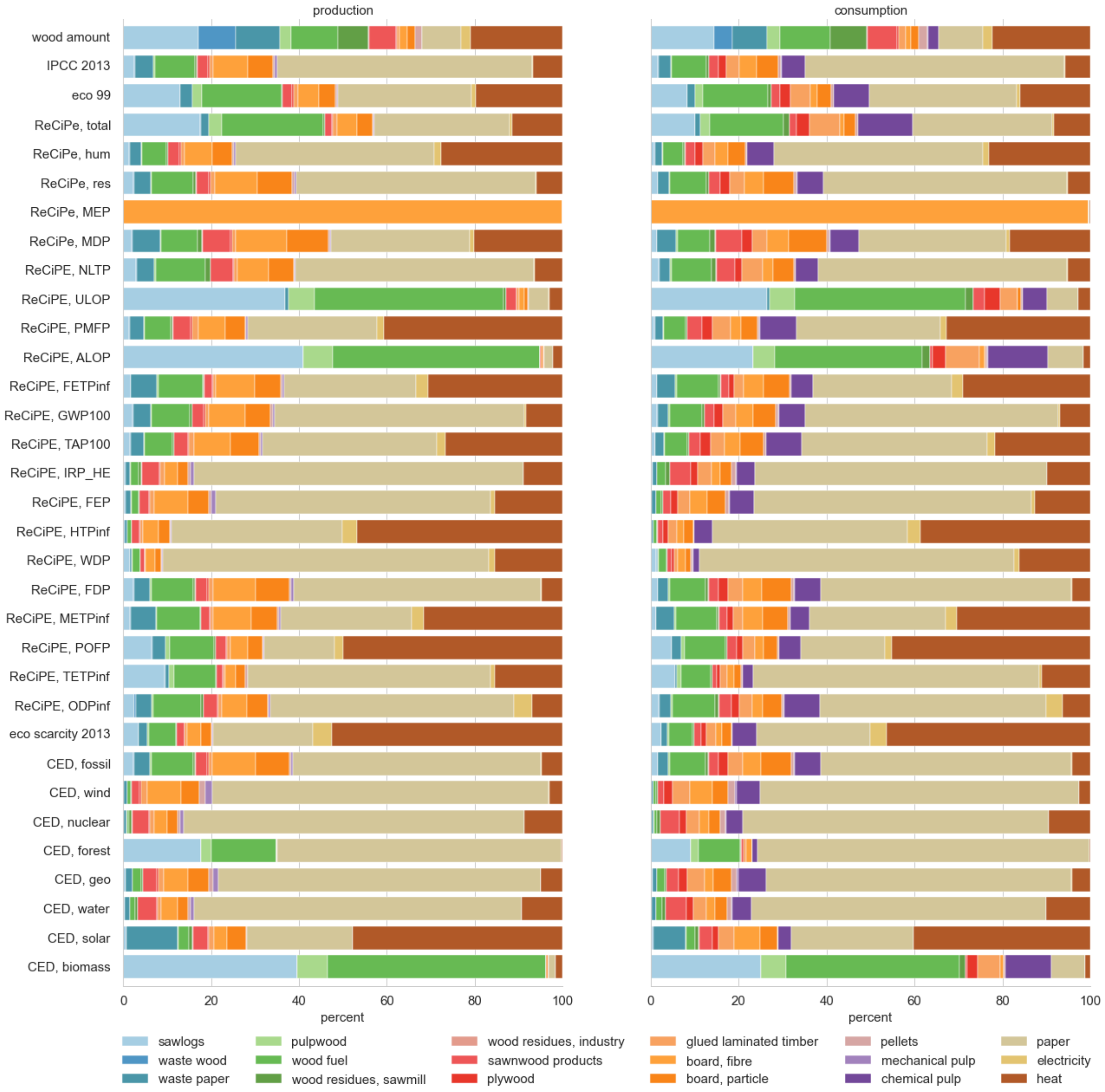

Figure S6: Impact share of the produced (left side) and consumed (right side) amount of wood in the different products. The impact shares are shown for different methods. Additionally, shares for the produced and consumed amount of wood in the different products are shown in the bar at the top of the figures (wood amount).

Impact share of primary wood

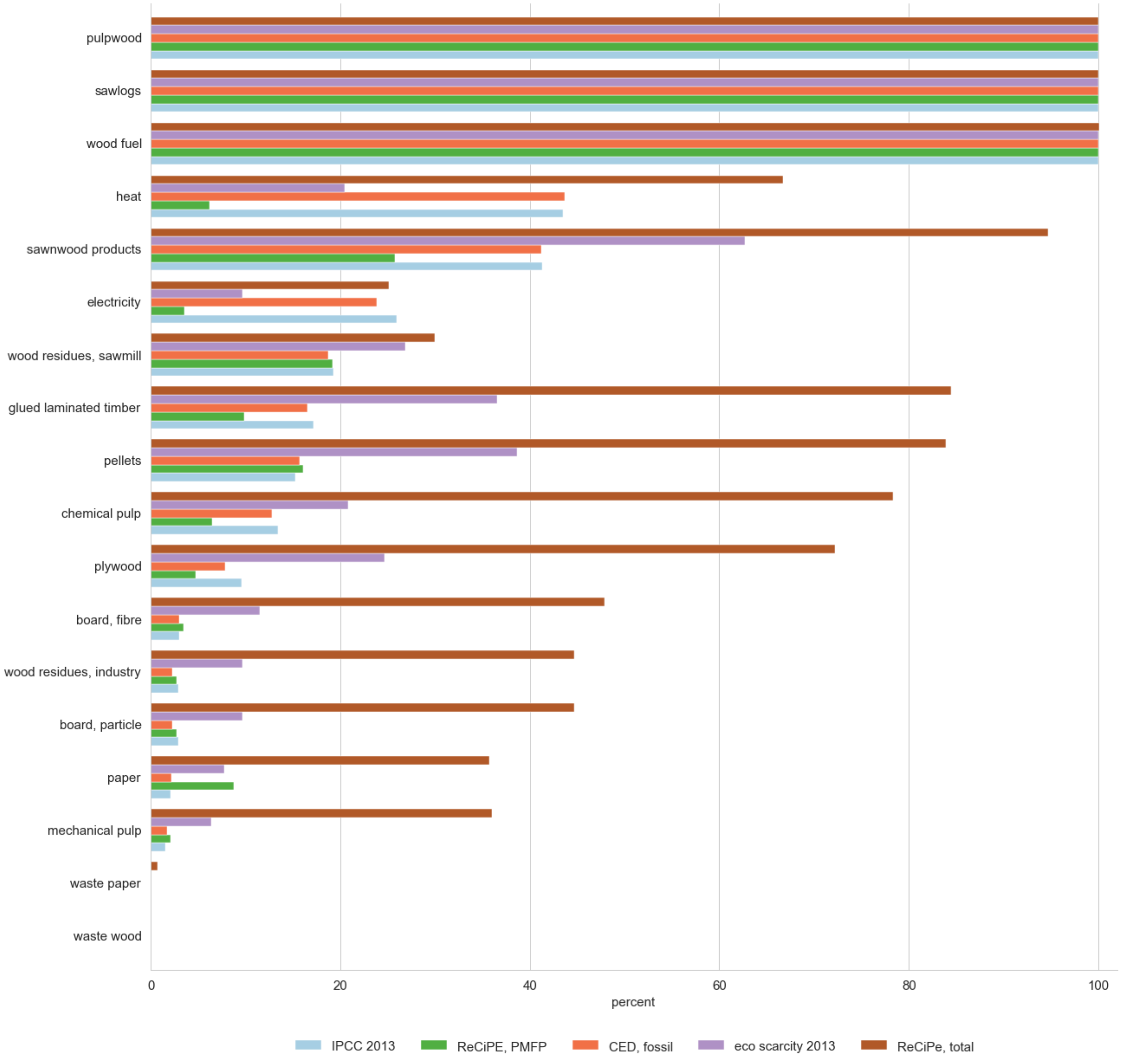

Figure S7: Impact share of primary wood provisioning on a products overall impact for the different products and impact methods. The share is calculated as impacts from primary wood provisioning up to the point of harvesting in relation to a products overall impacts.

Substitution effects

The subsequent part shows impacts and benefits of wood use compared to other materials. This comparison illustrates the environmental performance of wood. Thus, impacts from the production of non-wood products are accounted for as benefits (avoided impacts when using wood) whereas potential benefits from the end of life treatment of non-wood products are accounted for as impacts (unrealised benefits when using wood).

Results are shown for three scenarios, depending on the size of the expected profit when using wood (minimum, likely and maximum benefit). For each scenario a blue line indicates the cumulative total system impact/benefit when using wood instead of other materials.

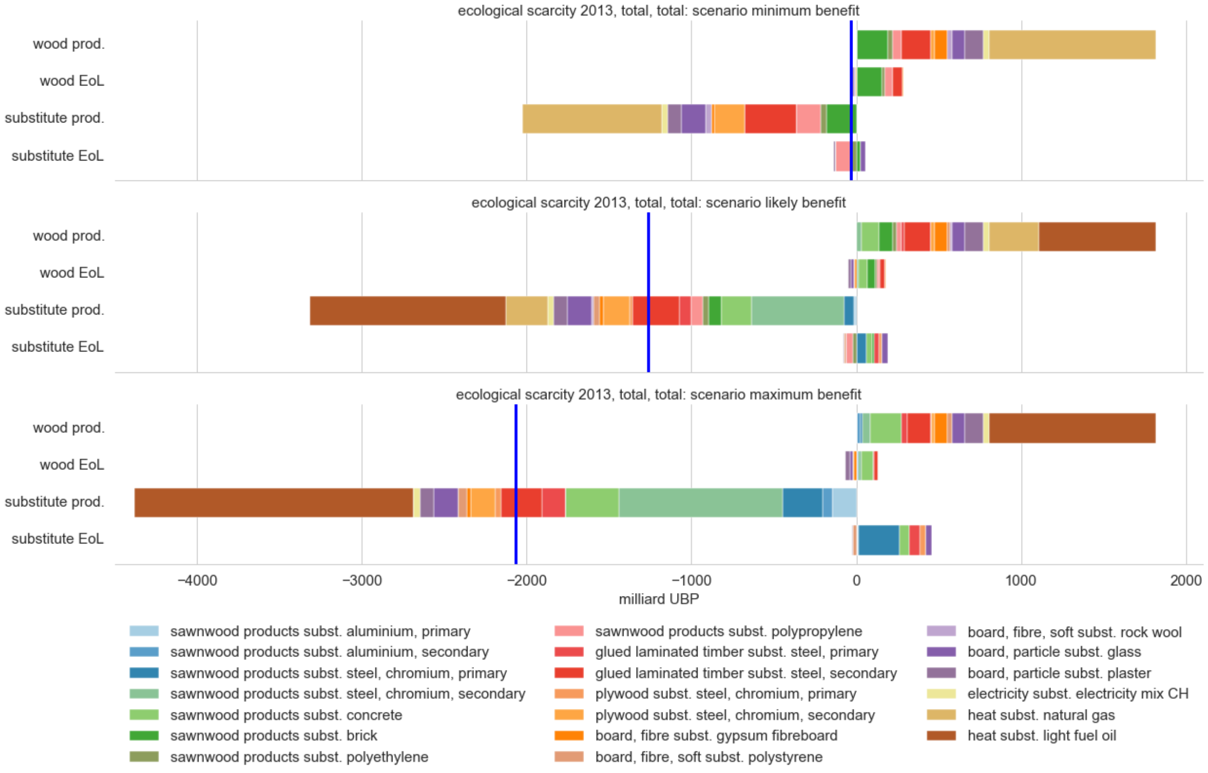

Figure S8: Environmental impacts (positive numbers) and benefits (negative numbers) from the production (prod.) and end of life (EoL) treatment of selected wood products and potential substitutes in UBP.

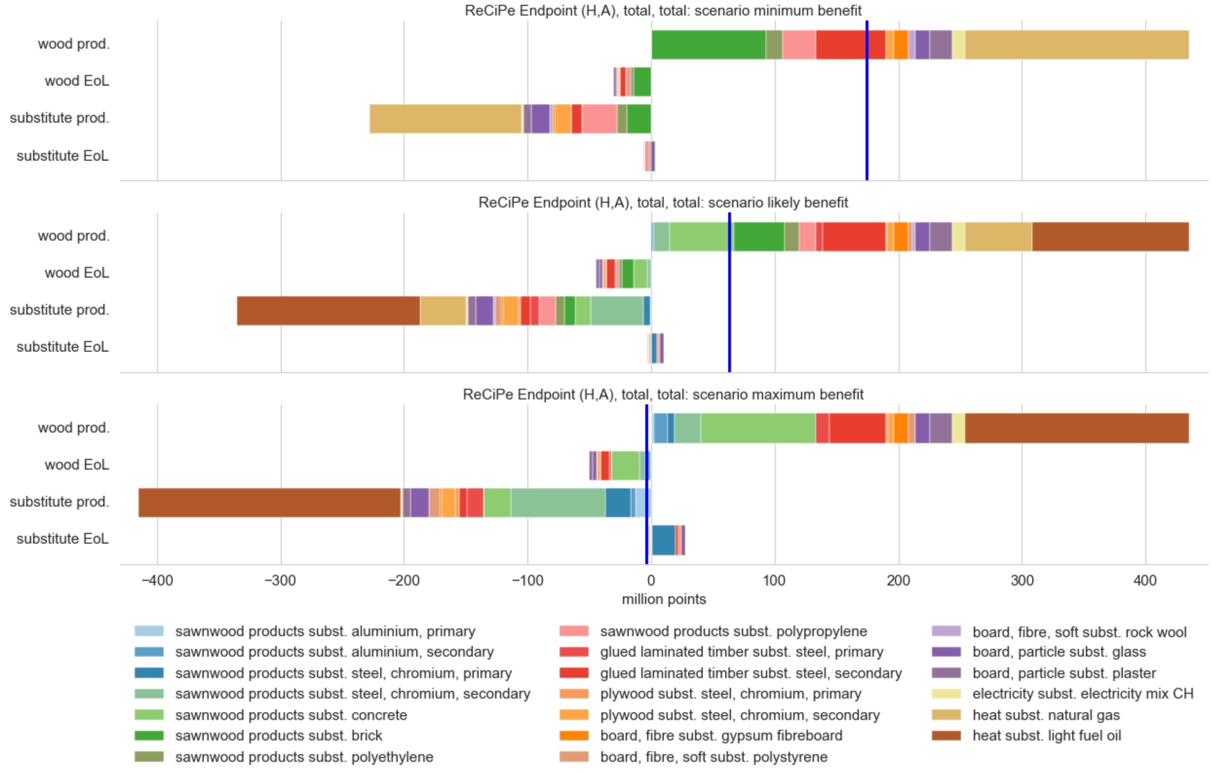

Figure S9: Environmental impacts (positive numbers) and benefits (negative numbers) from the production (prod.) and end of life (EoL) treatment of selected wood products and potential substitutes for the ReCiPe method.

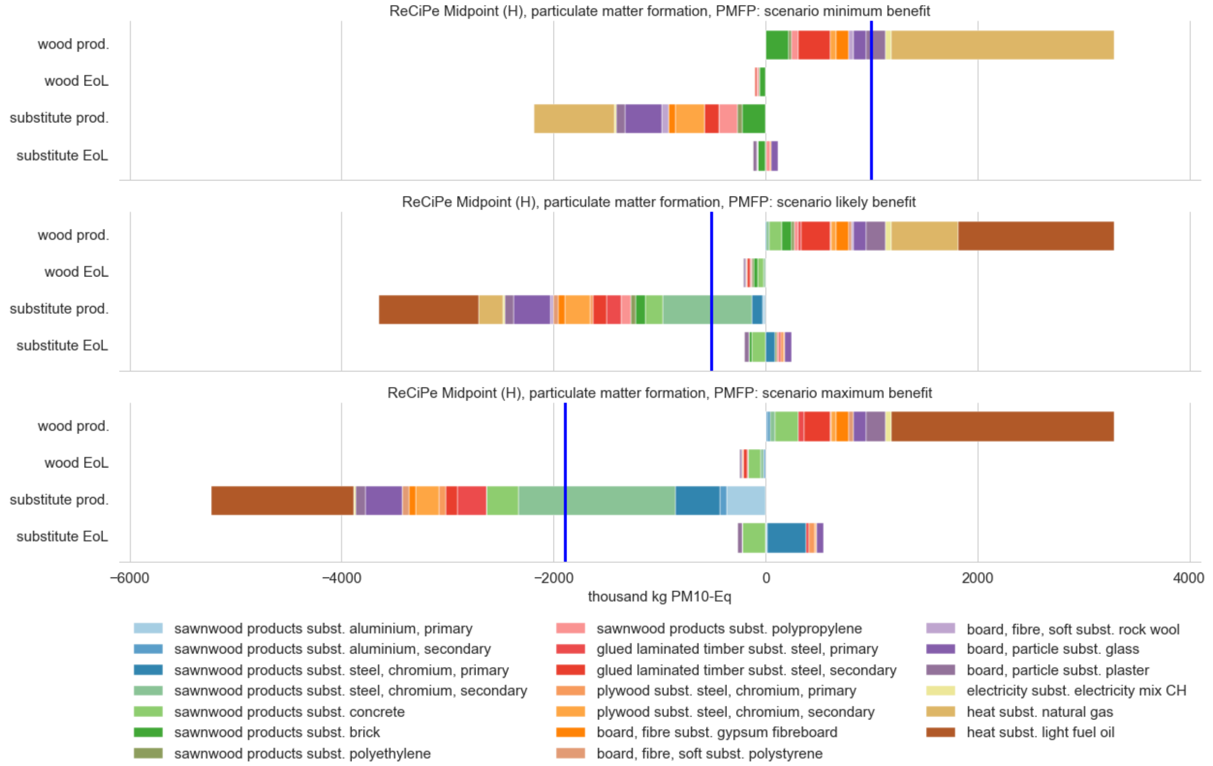

Figure S10: Environmental impacts (positive numbers) and benefits (negative numbers) from the production (prod.) and end of life (EoL) treatment of selected wood products and potential substitutes for the production of particulate matter.

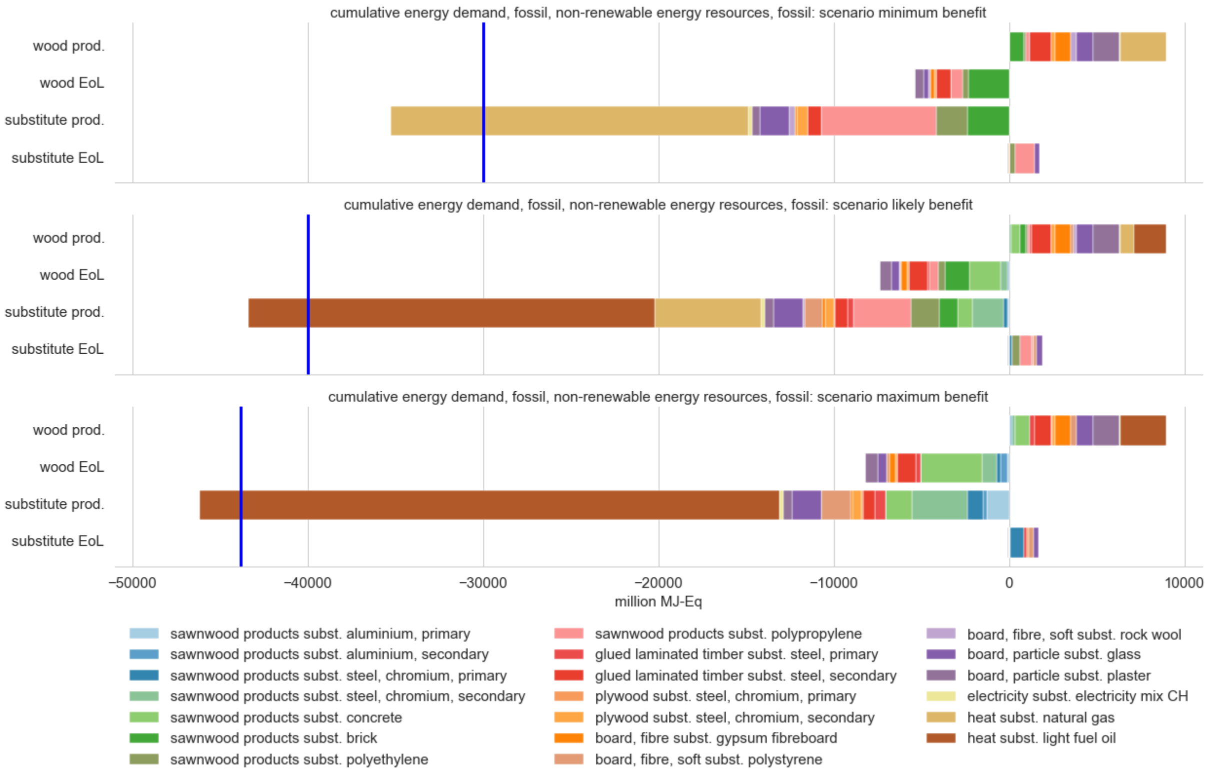

Figure S11: Environmental impacts (positive numbers) and benefits (negative numbers) from the production (prod.) and end of life (EoL) treatment of selected wood products and potential substitutes for the use of cumulative fossil energy demand.

Displacement factors for greenhouse gases

Table S3: Average reduction in climate change impacts per cubic meter of wood invested for the three different substitution scenarios per product. Numbers are given for benefits from material substitutions, energy substitutions and overall substitution. In each substitution case a distinction is made for displacement factors for production only and production including end of life. The average benefit represents the average over all three scenarios. Numbers are rounded.

			benefit per scenario and average across scenarios [t CO_2-eq/m^3 wood]							
			PRODUCTION ONLY				PRODUCTION AND END OF LIFE			
	wood	substitute	minimum benefit	likely benefit	maximum benefit	average	minimum benefit	likely benefit	maximum benefit	average
MATERIAL	glued laminated timber	steel, secondary	-0.06	-0.06	-0.06	-0.06	0.10	0.16	0.18	0.14
		steel, primary		0.58	0.58	0.58		0.67	0.69	0.68
	sawnwood products	concrete		0.27	0.27	0.27		0.49	0.51	0.50
		brick	0.18	0.18		0.18	0.34	0.40		0.37
		polyethylene	0.30	0.30		0.30	0.84	0.86		0.85
		aluminium, secondary		0.09	0.09	0.09		0.31	0.33	0.32
		aluminium, primary		3.67	3.67	3.67		3.75	3.77	3.76
		polypropylene	0.61	0.61		0.61	1.39	1.38		1.39
		steel, chromium, secondary		0.99	0.99	0.99		1.21	1.24	1.23
		steel, chromium, primary		1.23	1.23	1.23		0.40	0.42	0.41
	board, fibre	gypsum fibreboard	-0.44	-0.44	-0.44	-0.44	-0.36	-0.30	-0.27	-0.31
	board, fibre, soft	rock wool	-0.03	-0.03		-0.03	0.01	0.04		0.02
		polystyrene		0.55	0.55	0.55		1.07	1.07	1.07
	board, particle	plaster	0.06	0.06	0.06	0.06	0.13	0.18	0.21	0.17
		glass	0.41	0.41	0.41	0.41	0.25	0.30	0.32	0.29
	plywood	steel, chromium, secondary	1.22	1.22	1.22	1.22	1.45	1.54	1.57	1.52
		steel, chromium, primary		1.59	1.59	1.59		0.22	0.26	0.24
ENERGY	heat	natural gas	0.32	0.32		0.32	0.32	0.32		0.32
		light fuel oil		0.55	0.55	0.55		0.55	0.55	0.55
	electricity	electricity mix CH	0.12	0.12	0.12	0.12	0.12	0.12	0.12	0.12
	TOTAL		0.26	0.38	0.46	0.37	0.35	0.47	0.53	0.45

Data basis

Wood use

Modelled processes and products with clustering and ecoinvent activities

Table S4: List of modelled processes with name and corresponding product, affiliation to process-, product- and substitution cluster as well as corresponding ecoinvent activity (version 3.1 cut-off) at the tip of the respective process. Due to the modelling scheme in ecoinvent, part of the mechanical pulp production is already included in the paper processing activities.

PROCESS		MFA			ECOINVENT ACTIVITY AT TIP OF PROCESS		
Name	product	process cluster	product cluster	substitution cluster	process	product	geography
paper production, corrugated cardboard	corrugated cardboard	paper production	paper		corrugated board box production	corrugated board box	RER
sawmilling hardwood, bark chips	wood chips, wet	sawmilling	wood residues, sawmill		debarking, hardwood	bark chips, wet, measured as dry mass	CH
sawmilling softwood, bark chips	wood chips, wet	sawmilling	wood residues, sawmill		debarking, softwood	bark chips, wet, measured as dry mass	CH
board production, fibre, hard	hard board	board production	board, fibre	board, fibre	fibreboard production, hard	fibreboard, hard	RER
board production, fibre, soft	fibreboard, soft	board production	board, fibre	board, fibre, soft	fibreboard production, soft, from wet & dry processes	fibreboard, soft	Europe without Switzerland
paper production, folding boxboard	folding boxboard	paper production	paper		folding boxboard production	folding boxboard/chipboard	RER
glued laminated timber production	glued laminated timber	sawmilling	glued laminated timber	glued laminated timber	glued laminated timber production, for indoor use	glued laminated timber, for indoor use	RER
harvesting hardwood, fuel, chips	wood fuel, chips, hardwood	harvesting	wood fuel		hardwood forestry, mixed species, sustainable forest management	wood chips, wet, measured as dry mass	CH
harvesting hardwood, fuel, logs	wood fuel, logs, hardwood	harvesting	wood fuel		hardwood forestry, mixed species, sustainable forest management	cleft timber, measured as dry mass	CH

PROCESS		MFA			ECOINVENT ACTIVITY AT TIP OF PROCESS		
Name	product	process cluster	product cluster	substitution cluster	process	product	geography
harvesting hardwood, pulpwood	pulpwood, hardwood	harvesting	pulpwood		hardwood forestry, mixed species, sustainable forest management	pulpwood, hardwood, measured as solid wood under bark	CH
harvesting hardwood, sawlogs	sawlogs, hardwood	harvesting	sawlogs		hardwood forestry, mixed species, sustainable forest management	sawlog and veneer log, hardwood, measured as solid wood under bark	CH
incineration, special combustor, electricity	electricity	energy production	electricity	electricity	heat and power co-generation, wood chips, 6667 kW, state-of-the-art 2014	electricity, high voltage	CH
incineration, automatic firing	heat	energy production	heat	heat	heat production, hardwood chips from forest, at furnace 300kW, state-of-the-art 2014	heat, district or industrial, other than natural gas	CH
incineration, individual room heating	heat	energy production	heat	heat	heat production, mixed logs, at wood heater 6kW, state-of-the-art 2014	heat, central or small-scale, other than natural gas	CH
incineration, building heating	heat	energy production	heat	heat	heat production, wood pellet, at furnace 25kW, state-of-the-art 2014	heat, central or small-scale, other than natural gas	CH
paper production, kraft	kraft paper	paper production	paper		kraft paper production, unbleached	kraft paper, unbleached	RER
incineration, special combustor, heat	heat	energy production	heat	heat	market for heat, district or industrial, other than natural gas	heat, district or industrial, other than natural gas	CH
allocation, waste paper	waste paper	waste paper treatment	waste paper		market for waste paper, sorted	waste paper, sorted	GLO
allocation, waste wood	waste wood	waste wood treatment	waste wood		market for waste wood, post-consumer	waste wood, post-consumer	GLO
chipping, waste wood	wood chips, post-consumer wood	waste wood treatment	waste wood		market for wood chips, from post-consumer wood, measured as dry mass	wood chips, from post-consumer wood, measured as dry mass	GLO
board production, fibre, medium	medium density fibre board	board production	board, fibre	board, fibre	medium density fibre board production, uncoated	medium density fibreboard	RER
paper production, newsprint	newsprint paper	paper production	paper		paper production, newsprint, recycled	paper, newsprint	CH
paper production,	coated graphical	paper production	paper		paper production,	paper,	RER

**PROCESS**		**MFA**			**ECOINVENT ACTIVITY AT TIP OF PROCESS**		
**Name**	**product**	**process cluster**	**product cluster**	**substitution cluster**	**process**	**product**	**geography**
coated, wood containing	paper, wood containing				woodcontaining, lightweight coated	woodcontaining, lightweight coated	
paper production, uncoated, wood containing	uncoated graphical paper, wood containing	paper production	paper		paper production, woodcontaining, supercalendred	paper, woodcontaining, supercalendred	RER
paper production, coated, woodfree	coated graphical paper, woodfree	paper production	paper		paper production, woodfree, coated, at non-integrated mill	paper, woodfree, coated	RER
paper production, uncoated, woodfree	uncoated graphical paper, woodfree	paper production	paper		paper production, woodfree, uncoated, at non-integrated mill	paper, woodfree, uncoated	RER
board production, particle	particle board	board production	board, particle	board, particle	particle board production, uncoated, average glue mix	particleboard, uncoated	RER
wood residues production (proxy)	Proxy for)residues from industry) wood residues, dry	board production	wood residues, industry		particle board production, uncoated, average glue mix	residual wood, dry	RER
sawmilling hardwood, sawnwood, air drying	sawnwood, hardwood	sawmilling	sawnwood products	sawnwood products	planing, beam, hardwood, air dried	sawnwood, beam, hardwood, air dried, planed	CH
sawmilling hardwood, sawnwood, kiln drying	sawnwood, hardwood	sawmilling	sawnwood products	sawnwood products	planing, beam, hardwood, kiln dried	sawnwood, beam, hardwood, kiln dried, planed	CH
sawmilling softwood, sawnwood, air drying	sawnwood, softwood	sawmilling	sawnwood products	sawnwood products	planing, beam, softwood, air dried	sawnwood, beam, softwood, air dried, planed	CH
sawmilling softwood, sawnwood, kiln drying	sawnwood, softwood	sawmilling	sawnwood products	sawnwood products	planing, beam, softwood, kiln dried	sawnwood, beam, softwood, kiln dried, planed	CH
plywood production	plywood	sawmilling	plywood	plywood	plywood production, for outdoor use	plywood, for outdoor use	RER
sawmilling hardwood, wood residues	wood residues, hardwood, wet	sawmilling	wood residues, sawmill		sawing, hardwood	slab and siding, hardwood, wet, measured as dry mass	CH

PROCESS		MFA			ECOINVENT ACTIVITY AT TIP OF PROCESS		
**Name**	**product**	**process cluster**	**product cluster**	**substitution cluster**	**process**	**product**	**geography**
sawmilling softwood, wood residues	wood residues, softwood, wet	sawmilling	wood residues, sawmill		sawing, softwood	slab and siding, softwood, wet, measured as dry mass	CH
zz conversion, hardwood residues	wood residues, wet	sawmilling	wood residues, sawmill		slab and siding, hardwood, wet, measured as dry mass to generic market for residual hardwood, wet	residual hardwood, wet	GLO
zz conversion, softwood residues	wood residues, wet	sawmilling	wood residues, sawmill		slab and siding, softwood, wet, measured as dry mass to generic market for residual softwood, wet	residual softwood, wet	GLO
harvesting softwood, fuel, chips	wood fuel, chips, softwood	harvesting	wood fuel		softwood forestry, mixed species, sustainable forest management	wood chips, wet, measured as dry mass	CH
harvesting softwood, fuel, logs	wood fuel, logs, softwood	harvesting	wood fuel		softwood forestry, mixed species, sustainable forest management	cleft timber, measured as dry mass	CH
harvesting softwood, pulpwood	pulpwood, softwood	harvesting	pulpwood		softwood forestry, mixed species, sustainable forest management	pulpwood, softwood, measured as solid wood under bark	CH
harvesting softwood, sawlogs	sawlogs, softwood	harvesting	sawlogs		softwood forestry, mixed species, sustainable forest management	sawlog and veneer log, softwood, measured as solid wood under bark	CH
pulp production, chemical	sulfate pulp	pulp production	chemical pulp		sulfate pulp production, totally chlorine free bleached	sulfate pulp	RER
zz pulp production, mechanical	mechanical pulp	pulp production	mechanical pulp		thermo-mechanical pulp production	thermo-mechanical pulp	RER
paper production, tissue	tissue paper	paper production	paper		tissue paper production	tissue paper	RER
incineration, waste paper, electricity	electricity	energy production	electricity		treatment of waste graphical paper, municipal incineration	waste graphical paper	CH
incineration, waste paper, heat	heat	energy production	heat		treatment of waste graphical paper, municipal incineration	waste graphical paper	CH
zz pulp production, deinked	deinked pulp	pulp production	deinked pulp		treatment of waste paper to pulp, wet lap, totally chlorine	deinked pulp, wet lap	RoW

PROCESS		MFA			ECOINVENT ACTIVITY AT TIP OF PROCESS		
**Name**	**product**	**process cluster**	**product cluster**	**substitution cluster**	**process**	**product**	**geography**
					free bleached		
incineration, waste wood, electricity	electricity	energy production	electricity		treatment of waste wood, untreated, municipal incineration	waste wood, untreated	CH
incineration, waste wood, heat	heat	energy production	heat		treatment of waste wood, untreated, municipal incineration	waste wood, untreated	CH
zz chipping, hardwood residues, wet	wood chips, wet	sawmilling	wood residues, sawmill		wood chips production, hardwood, at sawmill	wood chips, wet, measured as dry mass	CH
zz chipping, softwood residues, wet	wood chips, wet	sawmilling	wood residues, sawmill		wood chips production, softwood, at sawmill	wood chips, wet, measured as dry mass	CH
zz chipping, wood residues, dry	wood chips, dry	board production	wood residues, industry		wood chips production, softwood, at sawmill	wood chips, wet, measured as dry mass	CH
pellet production	wood pellet	energy production	pellets		wood pellet production	wood pellet, measured as dry mass	RER

Units and conversion factors

Table S5: Processes with units and conversion factors. Since data is coming from different sources and is used in different systems, various units are used for the same product (m^3sw stands for m^3 solid wood equivalent). 'Ecoinvent' rely on ecoinvent 3.1 in the cut off version, 'statistics' rely on data from published statistics on domestic wood use, 'trade statistics' rely on data from published statistics on international wood trade, 'MFA' gives the unit for an MFA representation of the data. NaN means that no data was available. When simple mathematical conversions of units are not possible, the sources of conversion factors are indicated with numbers in the last column ('Source'): 1 Annual report on forest and wood from the Swiss Federal Office for the environment (BAFU 2012)**; 2 Ecoinvent database version 3.1** (Weidema et al. 2013)**; 3 Vademecum Holzenergie** (Holzenergie Schweiz 2008)**; 4 UNECE forest product conversion factors** (UNECE and FAO 2010)**; 5 Own assumptions. Numbers are rounded wherefore very small numbers appear as zero.**

PROCESS		UNITS				CONVERSION FACTORS						
name	product	ecoinvent	statistics	trade statistics	MFA	statistics to ecoinvent	trade statistics to ecoinvent	statistics to MFA	trade statistics to MFA	ecoinvent to MFA	MFA to ecoinvent	Source
board production, fibre, hard	hard board	m3	m3	t	m3sw	1.00	1.05	1.25	1.32	1.25	0.80	1, 2
board production, fibre, soft	fibreboard, soft	m3	m3	t	m3sw	1.00	6.29	0.56	2.24	0.56	1.79	1, 2
board production, fibre, medium	medium density fibre board	m3	m3	t	m3sw	1.00	1.46	1.24	1.55	1.24	0.81	1, 2
board production, particle	particle board	m3	m3	t	m3sw	1.00	1.57	0.94	1.44	0.94	1.06	1, 2
pulp production, chemical	sulfate pulp	kg	t	t	m3sw	1'000.00	1'000.00	1.80	1.80	0.00	555.56	1
zz pulp production, deinked	deinked pulp	kg	t	t	m3sw	1'000.00	1'000.00	1.80	1.80	0.00	555.56	1
incineration, special combustor, electricity	electricity	kWh	MWh	NaN	MWh	1'000.00	NaN	1.00	NaN	0.00	1'000.00	
incineration, waste wood, electricity	electricity	kWh	MWh	NaN	MWh	1'000.00	NaN	1.00	NaN	0.00	1'000.00	
incineration, waste paper, electricity	electricity	kWh	MWh	NaN	MWh	1'000.00	NaN	1.00	NaN	0.00	1'000.00	
glued laminated timber production	glued laminated timber	m3	m3sw	m3sw	m3sw	1.00	1.00	1.00	1.00	1.00	1.00	5
incineration, automatic firing	heat	MJ	MWh	NaN	MWh	3'600.00	NaN	1.00	NaN	0.00	3'600.00	
incineration, individual room heating	heat	MJ	MWh	NaN	MWh	3'600.00	NaN	1.00	NaN	0.00	3'600.00	

PROCESS		UNITS				CONVERSION FACTORS						
name	product	ecoinvent	statistics	trade statistics	MFA	statistics to ecoinvent	trade statistics to ecoinvent	statistics to MFA	trade statistics to MFA	ecoinvent to MFA	MFA to ecoinvent	Source
incineration, building heating	heat	MJ	MWh	NaN	MWh	3'600.00	NaN	1.00	NaN	0.00	3'600.00	
incineration, special combustor, heat	heat	MJ	MWh	NaN	MWh	3'600.00	NaN	1.00	NaN	0.00	3'600.00	
incineration, waste wood, heat	heat	MJ	MWh	NaN	MWh	3'600.00	NaN	1.00	NaN	0.00	3'600.00	
incineration, waste paper, heat	heat	MJ	MWh	NaN	MWh	3'600.00	NaN	1.00	NaN	0.00	3'600.00	
zz pulp production, mechanical	mechanical pulp	kg	t	t	m3sw	1'000.00	1'000.00	2.00	2.00	0.00	500.00	1
paper production, corrugated cardboard	corrugated cardboard	kg	t	t	m3sw	1'000.00	1'000.00	1.60	1.60	0.00	625.00	1
paper production, folding boxboard	folding boxboard	kg	t	t	m3sw	1'000.00	1'000.00	1.60	1.60	0.00	625.00	1
paper production, kraft	kraft paper	kg	t	t	m3sw	1'000.00	1'000.00	1.60	1.60	0.00	625.00	1
paper production, newsprint	newsprint paper	kg	t	t	m3sw	1'000.00	1'000.00	1.60	1.60	0.00	625.00	1
paper production, coated, wood containing	coated graphical paper, wood containing	kg	t	t	m3sw	1'000.00	1'000.00	1.60	1.60	0.00	625.00	1
paper production, uncoated, wood containing	uncoated graphical paper, wood containing	kg	t	t	m3sw	1'000.00	1'000.00	1.60	1.60	0.00	625.00	1
paper production, coated, woodfree	coated graphical paper, woodfree	kg	t	t	m3sw	1'000.00	1'000.00	1.60	1.60	0.00	625.00	1
paper production, uncoated, woodfree	uncoated graphical paper, woodfree	kg	t	t	m3sw	1'000.00	1'000.00	1.60	1.60	0.00	625.00	1
paper production, tissue	tissue paper	kg	t	t	m3sw	1'000.00	1'000.00	1.60	1.60	0.00	625.00	1
pellet production	wood pellet	kg	t	t	m3sw	1'000.00	1'000.00	2.28	2.28	0.00	438.60	3, 4
plywood production	plywood	m3	m3	t	m3sw	1.00	1.54	0.97	1.49	0.97	1.03	1
harvesting hardwood, pulpwood	pulpwood, hardwood	m3	m3sw	m3sw	m3sw	1.00	1.00	1.00	1.00	1.00	1.00	1
harvesting softwood, pulpwood	pulpwood, softwood	m3	m3sw	m3sw	m3sw	1.00	1.00	1.00	1.00	1.00	1.00	1

PROCESS		UNITS				CONVERSION FACTORS						
name	product	ecoinvent	statistics	trade statistics	MFA	statistics to ecoinvent	trade statistics to ecoinvent	statistics to MFA	trade statistics to MFA	ecoinvent to MFA	MFA to ecoinvent	Source
harvesting hardwood, sawlogs	sawlogs, hardwood	m3	m3sw	kg	m3sw	1.00	0.00	1.00	0.00	1.00	1.00	1
harvesting softwood, sawlogs	sawlogs, softwood	m3	m3sw	kg	m3sw	1.00	0.00	1.00	0.00	1.00	1.00	1
sawmilling hardwood, sawnwood, air drying	sawnwood, hardwood	m3	m3sw	kg	m3sw	1.00	0.00	1.00	0.00	1.00	1.00	1
sawmilling hardwood, sawnwood, kiln drying	sawnwood, hardwood	m3	m3sw	kg	m3sw	1.00	0.00	1.00	0.00	1.00	1.00	1
sawmilling softwood, sawnwood, air drying	sawnwood, softwood	m3	m3sw	kg	m3sw	1.00	0.00	1.00	0.00	1.00	1.00	1
sawmilling softwood, sawnwood, kiln drying	sawnwood, softwood	m3	m3sw	kg	m3sw	1.00	0.00	1.00	0.00	1.00	1.00	1
allocation, waste paper	waste paper	kg	t	t	m3sw	1'000.00	1'000.00	1.60	1.60	0.00	625.00	1
allocation, waste wood	waste wood	kg	t	t	m3sw	1'000.00	1'000.00	1.85	1.85	0.00	540.54	1
chipping, waste wood	wood chips, post-consumer wood	kg	kg	NaN	m3sw	909.09	NaN	0.00	NaN	0.00	909.09	1
harvesting hardwood, fuel, chips	wood fuel, chips, hardwood	kg	m3sw	kg	m3sw	909.09	1.00	1.00	0.00	0.00	909.09	1
harvesting hardwood, fuel, logs	wood fuel, logs, hardwood	kg	m3sw	m3sw	m3sw	900.90	900.90	1.00	1.00	0.00	900.90	1
harvesting softwood, fuel, chips	wood fuel, chips, softwood	kg	m3sw	kg	m3sw	909.09	1.00	1.00	0.00	0.00	909.09	1
harvesting softwood, fuel, logs	wood fuel, logs, softwood	kg	m3sw	m3sw	m3sw	900.90	900.90	1.00	1.00	0.00	900.90	1
wood residues production (proxy)	wood residues, dry	m3	m3sw	NaN	m3sw	1.11	1.11	1.00	1.00	0.90	1.11	1
zz chipping, wood residues, dry	wood chips, dry	kg	m3sw	NaN	m3sw	909.09	909.09	1.00	NaN	0.00	909.09	1
sawmilling hardwood, bark chips	wood chips, wet	kg	m3sw	NaN	m3sw	563.20	NaN	1.00	NaN	0.00	563.20	2
sawmilling softwood, bark chips	wood chips, wet	kg	m3sw	NaN	m3sw	387.20	NaN	1.00	NaN	0.00	387.20	2
sawmilling hardwood, wood residues	wood residues, hardwood, wet	kg	m3sw	m3sw	m3sw	719.42	719.42	1.00	1.00	0.00	719.42	1

PROCESS		UNITS				CONVERSION FACTORS						
name	product	ecoinvent	statistics	trade statistics	MFA	statistics to ecoinvent	trade statistics to ecoinvent	statistics to MFA	trade statistic to MFA	ecoinvent to MFA	MFA to ecoinvent	Source
sawmilling softwood, wood residues	wood residues, softwood, wet	kg	m3sw	m3sw	m3sw	719.42	719.42	1.00	1.00	0.00	719.42	1
zz conversion, hardwood residues	wood residues, wet	m3	m3sw	NaN	NaN	1.11	NaN	NaN	NaN	0.90	1.11	1
zz conversion, softwood residues	wood residues, wet	m3	m3sw	NaN	NaN	1.11	NaN	NaN	NaN	0.90	1.11	1
zz chipping, hardwood residues, wet	wood chips, wet	kg	NaN	NaN	NaN	NaN	NaN	NaN	NaN	NaN	NaN	
zz chipping, softwood residues, wet	wood chips, wet	kg	NaN	NaN	NaN	NaN	NaN	NaN	NaN	NaN	NaN	

Amounts

Table S6: Processes with product amounts for domestic production, import and export (respective units are given in Table S5). The table shows the amounts based on the statistics as well as for the MFA used as the basis for the study. Statistical data is coming from the following sources (indicated in the last column ('Source'): 1 Annual report on forest and wood from the Swiss Federal Office for the environment and respective background data as well as the Swiss statistics on wood use (BAFU 2012; BFS 2013)**; 2 Annual report of the Swiss paper producers** (ZPK 2011)**; 3 Annual statistics of the Swiss Federal Office for Energy and report about waste treatment in Switzerland** (BFE 2012a; BFE 2012b; BAFU and BFE 2014)**; 4 Industry analysis of the Swiss wood sector** (Lehner et al. 2014)**; 5 Own assumptions. The MFA data is based on the Swiss statistics of wood use** (BAFU 2012) **except for heat and electricity where data from the Swiss Federal Office for Energy and a report about waste treatment in Switzerland are used** (BFE 2012a; BFE 2012b; BAFU and BFE 2014) **NaN means that no data is available. Numbers are rounded.**

PROCESS		AMOUNT						
name	product	production statistics	import statistics	export statistics	production MFA	import MFA	export MFA	Source
board production, fibre, hard	hard board	0	35'064	147				1
board production, fibre, soft	fibreboard, soft	333'100	18'215	53'657	405'095	186'106	371'985	1
board production, fibre, medium	medium density fibre board	220'000	63'884	162'322				1
board production, particle	particle board	400'000	167'228	161'451	356'000	240'808	232'489	1
pulp production, chemical	sulfate pulp	0	299'743	24'591	0	496'296	6'415	1
zz pulp production, deinked	deinked pulp	758'000	6'295	76'715	NaN	NaN	NaN	2
incineration, special combustor, electricity	electricity	14'7996[1]	NaN	NaN	343'803	NaN	NaN	3
incineration, waste wood, electricity	electricity	195'807[2]	NaN	NaN				3
incineration, waste paper, electricity	electricity	168'275[3]	NaN	NaN	168'275	NaN	NaN	3
glued laminated timber production	glued laminated timber	120'000	227'424	NaN	120'000	227'424	NaN	4
incineration, automatic firing	heat	2'785'895[4]	NaN	NaN	7'441'849	NaN	NaN	3

PROCESS		AMOUNT						
**name**	**product**	**production statistics**	**import statistics**	**export statistics**	**production MFA**	**import MFA**	**export MFA**	**Source**
incineration, individual room heating	heat	1'310'858	NaN	NaN				3
incineration, building heating	heat	1'745'951	NaN	NaN				3
incineration, special combustor, heat	heat	797'845[5]	NaN	NaN				3
incineration, waste wood, heat	heat	801'301[6]	NaN	NaN				3
incineration, waste paper, heat	heat	276'790[3]	NaN	NaN	276'790	NaN	NaN	3
zz pulp production, mechanical	mechanical pulp	141'604	1'620	4'240	15'412	0	9'222	2
paper production, corrugated cardboard	corrugated cardboard	280'418	154'711	166'146	1'884'225	1'665'838	1'548'779	2
paper production, folding boxboard	folding boxboard	0	130'711	0				2
paper production, kraft	kraft paper	6'007	38'988	5'892				2
paper production, newsprint	newsprint paper	403'174	103'485	271'485				2
paper production, coated, wood containing	coated graphical paper, wood containing	164'321	96'867	138'265				2
paper production, uncoated, wood containing	uncoated graphical paper, wood containing	112'305	19'846	77'260				2
paper production, coated, woodfree	coated graphical paper, woodfree	146'285	133'192	133'028				2
paper production, uncoated, woodfree	uncoated graphical paper, woodfree	79'777	198'772	44'117				2
paper production, tissue	tissue paper	76'666	113'912	26'291				2
pellet production	wood pellet	145'000	39'000	5'000	330'600	88'920	11'400	4
plywood production	plywood	7'582	47'942	1'875	11'297	71'434	2'794	1

PROCESS		AMOUNT						
name	product	production statistics	import statistics	export statistics	production MFA	import MFA	export MFA	Source
harvesting hardwood, pulpwood	pulpwood, hardwood	237'028	0	47'000	548'039	151'305	108'610	1
harvesting softwood, pulpwood	pulpwood, softwood	296'450	151'000	62'000				1
harvesting hardwood, sawlogs	sawlogs, hardwood	296'352	42'709'640	183'987'504	3'625'135[7]	94'916[8]	816'877[8]	1
harvesting softwood, sawlogs	sawlogs, softwood	3'016'008	41'266'802	552'779'038				1
sawmilling hardwood, sawnwood, air drying	sawnwood, hardwood	41'796	25'500'476	8'457'095	1'198'000[9]	247'593[9]	222'542[9]	1
sawmilling hardwood, sawnwood, kiln drying	sawnwood, hardwood	20'895	12'748'325	4'227'913				1
sawmilling softwood, sawnwood, air drying	sawnwood, softwood	647'309	115'723'945	57'635'800				1
sawmilling softwood, sawnwood, kiln drying	sawnwood, softwood	545'009	97'434'914	48'527'029				1
allocation, waste paper	waste paper	1'315'470	257'658	572'163	2'104'752	412'253	915'461	2
allocation, waste wood	waste wood	993'123	0	551'004	1'837'277	0	1'019'357	1
chipping, waste wood	wood chips, post-consumer wood	0	NaN	NaN				1
harvesting hardwood, fuel, chips	wood fuel, chips, hardwood	488'347	9'623'631	38'118'137	2'296'755[10]	14'029[10]	18'145[10]	1
harvesting hardwood, fuel, logs	wood fuel, logs, hardwood	675'145	11'000	17'000				1
harvesting softwood, fuel, chips	wood fuel, chips, softwood	255'493	152'875'567	27'461'126				1
harvesting softwood, fuel, logs	wood fuel, logs, softwood	279'151	3'000	1'000				1
wood residues production (proxy)	wood residues, dry	47'305	NaN	NaN	54'731[11]	0	0	1

PROCESS		AMOUNT						
name	product	production statistics	import statistics	export statistics	production MFA	import MFA	export MFA	Source
zz chipping, wood residues, dry	wood chips, dry	NaN	NaN	NaN				5
sawmilling hardwood, bark chips	wood chips, wet	37'785	NaN	NaN	1'452'574[12]	425'648[12]	239'822[12]	1
sawmilling softwood, bark chips	wood chips, wet	448'277	NaN	NaN				1
sawmilling hardwood, wood residues	wood residues, hardwood, wet	39'284	20'551	5'859				1
sawmilling softwood, wood residues	wood residues, softwood, wet	775'714	390'849	111'441				1
zz conversion, hardwood residues	wood residues, wet	NaN	NaN	NaN				
zz conversion, softwood residues	wood residues, wet	NaN	NaN	NaN				
zz chipping, hardwood residues, wet	wood chips, wet	NaN	NaN	NaN				
zz chipping, softwood residues, wet	wood chips, wet	NaN	NaN	NaN				

[1] Produced electricity from wood cogen (cat. 18) plus electricity from non-waste wood share in renewable waste incineration (cat. 19)
[2] Produced electricity from municipal waste incineration (cat. 20) plus electricity from waste wood share in renewable waste incineration (cat. 19)
[3] Assumption: Waste paper is incinerated with a lower heating value of 14 GJ/t. Total energy content is then divided into electricity and heat according to efficiencies of municipal waste incineration in CH (25% heat, 15% electricity)
[4] Produced heat from automatic firing (cat. 12-18) minus heat from wood cogen (cat. 18) and minus heat produced within workshops (cat. 13, 15, 17), since internal heat production is already included in ecoinvent activities
[5] Produced heat from wood cogen (cat. 18) plus heat from non-waste wood share in renewable waste incineration (cat. 19)
[6] Produced heat from municipal waste incineration (cat. 20) plus heat from waste wood share in renewable waste incineration (cat. 19)
[7] Includes sawlogs, other (not defined) assortments and bark, since debarking happens at the sawmill in ecoinvent
[8] Includes sawlogs and other (not defined) assortments
[9] Includes sawnwood and veneer but excludes amounts of glued laminated timber and plywood (since they are considered separately)
[10] Includes wood fuel and 'Flurholz'
[11] Includes wood residues from industry without those for energy production (assumption: all energetically used wood residues from industry are used within workshops)
[12] Includes wood residues from sawmill plus bark (since debarking happens at the sawmill in ecoinvent). Assumption: Traded wood residues only originate from sawmills and not from industry, i.e. only wood residues, wet are traded

Conversion of energy to wood amounts

The following factors were used to convert energy amounts of wood incineration given in statistics to actual wood amounts incinerated. In the case of cogeneration of heat and electricity, a mass based allocation was used to split wood inputs accordingly.

Table S7: Conversion factors used to translate energy amounts into wood amounts. The last two columns give the factors for each energy system.

BFE Cat.[1]	ecoinvent process	Geography	Heat efficiency[2]	Electrical efficiency[2]	Final energy 2011[3]	Wood input[3]	Useful energy	Wood input / useful heat	Wood input / useful electricity
			%	%	MWh	m3	MWh	m3/MWh	m3/MWh
1-6	heat production, mixed logs, at wood heater 6kW, state-of-the-art 2014	CH	60	0	2’304’665	810’480	1’382’799	0.59	0
7-11	heat production, wood pellet, at furnace 25kW, state-of-the-art 2014	CH	80	0	2’473’850	904’313	1’979’080	0.46	0
12-17	heat production, hardwood chips from forest, at furnace 300kW, state-of-the-art 2014	CH	80	0	3’703’745	1’431’346	2’962’996	0.48	0
18	heat and power co-generation, wood chips, 6667 kW, state-of-the-art 2014	CH	45	15	1’081’406	449’556	648’844	0.69	0.69
19-20	treatment of waste wood, untreated, municipal incineration	CH	25	15	2’483’529	927’269	993’412	0.93	0.93
-	treatment of waste graphical paper, municipal incineration	CH	25	15	1’100’644	452’800	440’257	1.03	1.03

[1] Categories used in the statistics of Bundesamt für Energie (BFE) to categorize incineration systems
[2] Efficiencies from ecoinvent and Swiss averages
[3] Numbers from statistics of the Bundesamt für Energie (BFE)

Substitution

Substitution products, factors and ecoinvent activities

Table S8: Wood products and their substitution products, including substitution factors and respective ecoinvent activities in the version 3.1 (cut-off) used.

MFA substitution cluster	unit	substitution product	unit	service	substitution factor	substitute ecoinvent process	substitute ecoinvent product	substitute geography
glued laminated timber	m3	steel, secondary	kg	construction	369.50	steel production, electric, low-alloyed	steel, low-alloyed	RER
glued laminated timber	m3	steel, primary	kg	construction	369.50	Steel production, converter, low-alloyed	Steel, low-alloyed	RER
sawnwood products	m3	concrete	m3	construction	1.20	concrete production, normal	concrete, normal	CH
sawnwood products	m3	brick	kg	construction	1'024.50	brick production	brick	RER
sawnwood products	m3	polyethylene	kg	packaging	187.50	polyethylene production, high density, granulate	polyethylene, high density, granulate	RER
sawnwood products	m3	aluminium, secondary	kg	packaging	241.07	treatment of aluminium scrap, post-consumer, prepared for recycling, at remelter	aluminium, wrought alloy	GLO
sawnwood products	m3	aluminium, primary	kg	packaging	241.07	aluminium ingot, primary, to aluminium, wrought alloy market	aluminium, wrought alloy	RER
sawnwood products	m3	polypropylene	kg	furniture	348.66	polypropylene production, granulate	polypropylene, granulate	RER
sawnwood products	m3	steel, chromium, secondary	kg	furniture	321.89	steel production, electric, chromium steel 18/8	steel, chromium steel 18/8	RER
sawnwood products	m3	Steel, chromium, primary	kg	furniture	321.89	steel production, converter, chromium steel 18/8	Steel, chromium steel 18/8	RER
board, fibre	m3	gypsum fibreboard	kg	construction	1'366.67	gypsum fibreboard production	gypsum fibreboard	CH
board, fibre	m3	*none*		furniture				
board, fibre, soft	m3	rock wool	kg	construction	132.50	rock wool production	rock wool	CH
board, fibre, soft	m3	polystyrene	kg	construction	128.71	polystyrene foam slab production, 10% recycled	polystyrene foam slab, 10% recycled	CH
board, particle	m3	plaster	kg	construction	1'566.39	base plaster production	base plaster	CH
board, particle	m3	glass	kg	furniture	892.89	flat glass production, uncoated	flat glass, uncoated	RER

plywood	m3	*none*		construction				
plywood	m3	steel, chromium, primary	kg	furniture	503.47	steel production, electric, chromium steel 18/8	steel, chromium steel 18/8	RER
plywood	m3	steel, chromium, primary	kg	furniture	503.47	steel production, converter, chromium steel 18/8	steel, chromium steel 18/8	RER
heat	MJ	natural gas	MJ	heat	1.00	market for heat, district or industrial, natural gas	heat, district or industrial, natural gas	CH
heat	MJ	light fuel oil	MJ	heat	1.00	heat production, light fuel oil, at boiler 10kW, non-modulating	heat, central or small-scale, other than natural gas	CH
electricity	kWh	electricity mix CH	kWh	electricity	1.00	market for electricity, medium voltage	electricity, medium voltage	CH
paper	kg	*none*		print				

Substitution parameters

Table S9: Parameters used for the calculation of the substitution factors.

parameters	amount	unit	function	source	comment
concrete in wall	371.8	kg/m2	support	Bauteilkatalog (BFE-Kataloge): W31	
sawmwood in wall	65.3	kg/m2	support	Bauteilkatalog (BFE-Kataloge): W47i	Blockwand (Massivholz) + Lattenrost + Holzlatte
brick in wall	133.8	kg/m2	support	Bauteilkatalog (BFE-Kataloge): W25	
gypsum plasterboard in wall	10.6	kg/m2	covering	Bauteilkatalog (BFE-Kataloge): W38	
sawnwood (Täfer) in wall	6.1	kg/m2	covering	Bauteilkatalog (BFE-Kataloge): W34	assumption: same for particle board
plaster in wall	15	kg/m2	covering	Bauteilkatalog (BFE-Kataloge): W01	
fibreboard in wall	9	kg/m2	covering	Bauteilkatalog (BFE-Kataloge): Wi101	assumption: used in the same amount for covering
particle board in furniture	23.4	kg/p	table leaf	IKEA Torsby Tisch mit Holzplatte	Verpackungsmasse
glass in furniture	32.8	kg/p	table leaf	IKEA Torsby Tisch mit Glasplatte	Verpackungsmasse
plywood in furniture	7.2	kg/p	shelf	IKEA Albert Regal	Verpackungsmasse
steel in furniture	5.8	kg/p	shelf	IKEA Hyllis Regal	Verpackungsmasse
sawnwood in furniture	0.0127	m3/p	chair	Krasnobaev 2013 (Projektarbeit)	
polypropylene in furniture	4.428	kg/p	chair	Krasnobaev 2013 (Projektarbeit)	assumption: all plastic is polypropylene
sawnwood in packaging	28	kg/p	pallet	udobaer.ch	EUR-Tauschpalette
polyethylene in packaging	10.5	kg/p	pallet	udobaer.ch	Displaypalette (ungefähr gleiche Traglast wie EUR-Palette)
fibreboard (LDF) thermal conductivity	0.042	W/(mK)	insulation	Gebäudehülle Schweiz 2011	assumption: 17% more material is needed for same thermal conductivity as rock wool
rock wool thermal conductivity	0.035	W/(mK)	Insulation	Gebäudehülle Schweiz 2011	thermal conductivity of rock wool approximately 17% better than for LDF
average density CH wood	500	kg/m3		BAFU (2007): CO2-Effekte der Schweizer Wald- und Holzwirtschaft	
average density particle board	637	kg/m3		ecoinvent	
average density fibreboard (HDF and MDF)	820	kg/m3		ecoinvent	
average density fibreboard (LDF)	159	kg/m3		ecoinvent	
average density plywood	625	kg/m3		ecoinvent	
average density concrete	2380	kg/m3		ecoinvent	

End of Life

End of Life parameters

Table S10: Parameters used for the calculation of end of life environmental impacts and benefits.

parameter	amount	unit	source	comments
MSWI electric efficiency CH	0.15	%	BFE (2013): Einheitliche Heizwert- und Energiekennzahlenberechnung der Schweizer KVA nach europäischem Standardverfahren (Resultate 2012)	Swiss average
MSWI heat efficiency CH	0.25	%	BFE (2013): Einheitliche Heizwert- und Energiekennzahlenberechnung der Schweizer KVA nach europäischem Standardverfahren (Resultate 2012)	Swiss average
Cogen for renewable waste electric efficiency CH	0.15	%	BFE (2012): Teilstatistik Spezielle energetische Holznutzung: Erhebungsjahr 2011	Swiss average
Cogen for renewable waste heat efficiency CH	0.45	%	BFE (2012): Teilstatistik Spezielle energetische Holznutzung: Erhebungsjahr 2011	Swiss average
average density of wood	500	kg/m3	BAFU (2007): CO2-Effekte der Schweizer Wald- und Holzwirtschaft	
average density concrete	2380	kg/m3	ecoinvent	
average density board, fibre	820	kg/m3	ecoinvent	average from MDF and HDF
average density board, fibre, soft	159	kg/m3	ecoinvent	
average density board, particle	637	kg/m3	ecoinvent	
average density plywood	489.22	kg/m3	Rüter & Diederichs (2012): Ökobilanz Basisdaten für Bauprodukte aus Holz	Brettsperrholz
average density glued laminated timber	507.11	kg/m3	Rüter & Diederichs (2012): Ökobilanz Basisdaten für Bauprodukte aus Holz	Brettschichtholz, Standardträger
average wood content board, fibre	711.2	kg/m3	ecoinvent	average from MDF and HDF
average wood content board, fibre, soft	140.8	kg/m3	ecoinvent	
average wood content board, particle	535.8	kg/m3	ecoinvent	
average wood content plywood	430.23	kg/m3	Rüter & Diederichs (2012): Ökobilanz Basisdaten für Bauprodukte aus Holz	Brettsperrholz

parameter	amount	unit	source	comments
average wood content glued laminated timber	444.91	kg/m3	Rüter & Diederichs (2012): Ökobilanz Basisdaten für Bauprodukte aus Holz	Brettschichtholz, Standardträger
lower heating value waste wood	13.99	MJ/kg	ecoinvent	used for all waste wood coming from the different products
lower heating value polyethylene	42.47	MJ/kg	ecoinvent	
lower heating value polypropylene	32.78	MJ/kg	ecoinvent	
lower heating value polystyrene	38.67	MJ/kg	ecoinvent	

End of Life ecoinvent activities

Table S11: Products with respective ecoinvent activities used for the calculation of end of life treatment impacts in recycling, landfilling or incineration as well as activities representing the replaced product. Products are only listed when landfilling, incineration or recycling (without recycled content from cut-off) exist as end of life options.

Product	Part	Ecoinvent process	Ecoinvent product	Ecoinvent geography
steel, primary	treatment recycling	treatment of waste bulk iron, excluding reinforcement, sorting plant	waste bulk iron, excluding reinforcement	CH
	replaced product	steel production, electric, low-alloyed	Steel, low-alloyed	RER
steel, chromium, primary	treatment recycling	treatment of waste bulk iron, excluding reinforcement, sorting plant	waste bulk iron, excluding reinforcement	CH
	replaced product	steel production, electric, chromium steel 18/8	steel, chromium steel 18/8	RER
aluminium, primary	treatment recycling	treatment of waste bulk iron, excluding reinforcement, sorting plant	waste bulk iron, excluding reinforcement	CH
	replaced product	treatment of aluminium scrap, post-consumer, prepared for recycling, at remelter	aluminium, wrought alloy	RER
concrete	treatment recycling	treatment of waste concrete, not reinforced, recycling	waste concrete, not reinforced	CH
	treatment landfilling	treatment of waste concrete, not reinforced, collection for final disposal	waste concrete, not reinforced	CH
	replaced product	gravel production, crushed	gravel, crushed	CH
polyethylene	treatment recycling	treatment of waste concrete, not reinforced, recycling	waste concrete, not reinforced	CH
	treatment incineration	treatment of waste polyethylene, municipal incineration	waste polyethylene	CH
	replaced product	ethylene production, average	ethylene, average	RER
polypropylene	treatment recycling	treatment of waste concrete, not reinforced, recycling	waste concrete, not reinforced	CH
	treatment incineration	treatment of waste polypropylene, municipal incineration	waste polypropylene	CH
	replaced product	propylene production	propylene	RER
brick	treatment recycling	treatment of waste brick, recycling	waste brick	CH
	replaced product	gravel production, crushed	gravel, crushed	CH
glass[1]	treatment recycling	treatment of waste glass from unsorted public collection, sorting	glass cullet, sorted	RER
	treatment landfilling	treatment of waste glass, inert material landfill	waste glass	CH
	replaced product	foam glass production	foam glass	GLO
		foam glass production, without cullet	foam glass	GLO
gypsum fibreboard	treatment recycling	treatment of waste gypsum plasterboard, recycling	waste gypsum plasterboard	CH

	treatment landfilling	treatment of waste gypsum plasterboard, collection for final disposal	waste gypsum plasterboard	CH
	replaced product	gypsum quarry operation	gypsum, mineral	CH
rock wool[2]	treatment landfilling	treatment of waste mineral wool, collection for final disposal	waste mineral wool	CH
polystyrene	treatment incineration	treatment of waste polystyrene, municipal incineration	waste polystyrene	CH
plaster	treatment landfilling	treatment of waste mineral plaster, collection for final disposal	waste mineral plaster	CH
glued laminated timber	treatment energy	treatment of waste wood, untreated, municipal incineration	waste wood, untreated	CH
sawnwood products	treatment energy	treatment of waste wood, untreated, municipal incineration	waste wood, untreated	CH
board, fibre	treatment energy	treatment of waste fibreboard, collection for final disposal	waste fibreboard	CH
board, fibre, soft	treatment energy	treatment of waste fibreboard, collection for final disposal	waste fibreboard	CH
board, particle	treatment energy	treatment of waste fibreboard, collection for final disposal	waste fibreboard	CH
plywood	treatment energy	treatment of waste wood, untreated, municipal incineration	waste wood, untreated	CH

[1] For glass the replaced product is the difference between foam glass without and with glass cullet, since most of the recycled flat glass is used in foam glass.
[2] Large amounts of rock wool go into municipal waste incineration and ends up in the sludge (since incombustible). The sludge goes then to landfill.

References

BAFU (2012) Jahrbuch Wald und Holz. [Swiss statistical yearbook of forestry]. Bern: BAFU, Federal Office for the Environment

BAFU, BFE (2014) Transformation der Abfallverwertung in der Schweiz für eine hohe und zeitlich optimierte Energieausnutzung. [Transformation of waste recycling in Switzerland for a high and temporally optimized energy utilization]. Bern: BAFU, Federal Office for the Environment; BFE, Federal Office of Energy

BFE (2012a) Schweizerische Holzenergiestatistik - Erhebung für das Jahr 2011. [Swiss wood energy statistics - Survey for 2011]. Bern: BFE, Federal Office of Energy

BFE (2012b) Teilstatistik Spezielle energetische Holznutzungen : Feuerungen und Motoren für erneuerbare Abfälle Erhebungsjahr 2011. [Part statistic special energy usages of wood: furnaces and engines for renewable waste collection 2011]. Bern: BFE, Federal Office of Energy

BFS (2013) Holzverarbeitungserhebung. [Wood-working survey]. http://www.bfs.admin.ch/bfs/portal/de/index/themen/07/01/new/nip_detail.html?gnpID=2013-413. Accessed 29 May 2015

Holzenergie Schweiz (2008) Vademecum Holzenergie. [Vademecum Wood energy]. Zürich

Lehner L, Kinnunen H, Weidner U, et al (2014) Branchenanalyse - Analyse und Synthese der Wertschöpfungskette (WSK) Wald und Holz in der Schweiz. [Industry analysis - analysis and synthesis of the value chain (WSK) forest and wood in Switzerland]. Abensberg

UNECE, FAO (2010) Geneva Timber and Forest Discussion Paper 49: Forest Product Conversion Factors for the UNECE Region. Geneva: UNECE, United Nations Economic Commission for Europe; FAO, Food and Agriculture Organization of the United Nations

Weidema BP, Bauer C, Hischier R, et al (2013) Overview and methodology. Data quality guideline for the ecoinvent database version 3. St. Gallen

ZPK (2011) Jahresbericht 2011. [Annual report 2011]. Zurich: ZPK, Verband der Schweizerischen Zellstoff-, Papier- und Kartonindustrie
